# DMAMCL induces ferroptosis in neuroblastoma by targeting HMOX1 in MYCN-amplified subtypes whereas targeting STEAP3 in MYCN-nonamplified subtypes

**DOI:** 10.1080/13510002.2026.2702136

**Published:** 2026-07-13

**Authors:** Yifan Ma, Simeng Zhang, Dongyang Zhang, Zhongyan Hua, Zhijie Li

**Affiliations:** a Department of Pediatrics, Shengjing Hospital of China Medical University, Shenyang, China; b Medical Research Center, Liaoning Key Laboratory of Research and Application of Animal Models for Environment and Metabolic Diseases, Shengjing Hospital of China Medical University, Shenyang, China

**Keywords:** Neuroblastoma, DMAMCL, ferroptosis, HMOX1, MYCN, STEAP3, KEAP1, NRF2

## Abstract

**Objectives:**

Neuroblastoma (NB) is a common pediatric extracranial solid tumor. Dimethylaminomicheliolide (DMAMCL), a prodrug of Micheliolide (MCL), shows antitumor activity against NB, but its mechanisms remain unclear. This study investigated the antitumor mechanisms of DMAMCL in NB.

**Methods:**

Key pathways/genes were identified by RNA-seq and ferroptosis PCR array. Ferroptosis was confirmed by indicators. Mechanisms were investigated using siRNAs, shRNAs, and overexpression plasmids *in vitro* and *in vivo*. Direct targets were screened by LiP-MS. Molecular biology experiments elucidated the mechanisms.

**Results:**

DMAMCL induced ferroptosis in NB *in vitro* and *in vivo*, upregulating *HMOX1*. *HMOX1* knockdown attenuated DMAMCL-induced ferroptosis and its overexpression triggered ferroptosis in *MYCN*-amplified NB cells but not in *MYCN*-nonamplified cells. DMAMCL-induced ferroptosis via *HMOX1* upregulation depended on MYCN levels. Mechanistically, DMAMCL bound to KEAP1 in *MYCN*-amplified NB cells, increasing nuclear NRF2 and upregulating HMOX1. In *MYCN-*nonamplified NB cells, DMAMCL upregulated STEAP3, increasing Fe^2+^ and lipid peroxidation to induce ferroptosis. STEAP3 overexpression induced ferroptosis and suppressed tumor growth.

**Disscusion:**

DMAMCL induces ferroptosis in NB through MYCN-associated dual pathways, activating the NRF2/HMOX1 axis via KEAP1 binding in *MYCN*-amplified cells, while upregulating STEAP3 in *MYCN*-nonamplified cells. This provides new insights for DMAMCL application in treating NB subtypes with different MYCN levels.

## Graphical abstract

**Figure:**
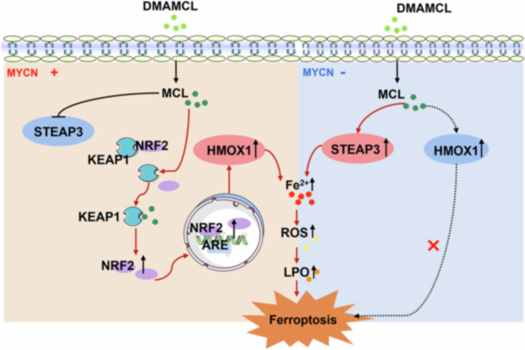

## 1. Introduction

Neuroblastoma (NB) is a malignant embryonal tumor arising from neural crest derivatives and is the most prevalent extracranial solid malignancy in the pediatric population [1]. NB accounts for 7% of all pediatric cancers and 15% of cancer-related deaths in children [2,3]. NB were stratified into low-, intermediate-, and high-risk categories. Patients with low- and intermediate-risk disease demonstrate favorable prognoses, with a five-year survival rate exceeding 90% [4]. Despite multimodal therapeutic approaches, the five-year survival rate of patients with high-risk NB (HR-NB) remains below 50% [5]. Patients with HR-NB show genetic features in major risk biomarkers, such as *MYCN* amplification [6]. Developing effective therapeutic strategies to improve clinical outcomes in patients with HR-NB remains a critical challenge in basic research and clinical practice. Current treatment modalities demonstrate limited efficacy in overcoming chemotherapy resistance and preventing disease relapse, highlighting the urgent need for novel therapeutic agents with improved clinical applicability to this aggressive malignancy.

Micheliolide (MCL) is a natural guaianolide sesquiterpene lactone isolated from Michelia compressa and M. champaca. DMAMCL is a water-soluble prodrug of MCL. Under physiological conditions, such as pH 7.4 HEPES buffer or plasma, DMAMCL can be consistently and steadily converted into its active metabolite MCL [7]. DMAMCL was developed to possess superior stability and activity, as well as reduced toxicity in normal and stem cells relative to MCL [8–10]. Studies have reported that DMAMCL has anti-tumor effects in various tumors, such as hepatocellular carcinoma, glioblastoma, and rhabdomyosarcoma [11–13]. Furthermore, DMAMCL has entered phase II clinical trials for glioblastoma treatment in China and Australia, demonstrating its translational potential in neuro-oncology [14]. We previously reported that DMAMCL had a significant anti-tumor effect on NB *in vitro* and *in vivo* [15]. However, whether other molecular mechanisms are involved in DMAMCL’s anti-NB effects requires further study.

Ferroptosis is an iron-dependent cell death characterized by excessive accumulation of lipid peroxides [16]. Disruption of iron metabolism and dysfunctional glutathione peroxidase 4 (GPX4)-mediated lipid peroxidation scavenging are central to the pathogenesis of ferroptosis [17]. The iron redox cycle affects the sensitivity of cells to ferroptosis [18]. This process typically involves extracellular iron uptake, intracellular ferrous iron generation, and subsequent ion mobilization [19]. For example, Fe^3+^ is transported into cells via the membrane protein transferrin receptor 1 and reduced to Fe^2+^ by STEAP3 [20,21]. HMOX1 is a key factor regulating iron metabolism in cells as an enzyme, and it catalyzes heme degradation and releases Fe^2+^ [22,23]. Emerging evidence reveals that ferroptosis plays a key role in tumor therapy. The metabolic reprogramming inherent to cancer cells, marked by elevated ROS and enriched polyunsaturated fatty acid pools, confers selective susceptibility to ferroptosis in tumors [24]. Numerous studies have demonstrated that ferroptosis inducers not only significantly inhibit NB progression but also sensitize tumor cells to chemotherapeutic agents such as etoposide [25–27], suggesting that ferroptosis induction may represent a promising therapeutic strategy against NB.

This study explored the antitumor mechanisms of DMAMCL in NB. By using RNA-seq, functional assays and mechanistic investigations, we identified that DMAMCL induced ferroptosis in NB, and the key molecular target that mediates DMAMCL-induced ferroptosis differs between *MYCN*-amplified and *MYCN*-nonamplified NB cells. DMAMCL bound to KEAP1, and this binding increased nuclear NRF2 levels and upregulated *HMOX1* expression, thereby inducing ferroptosis in *MYCN*-amplified NB cells. However, in *MYCN*-nonamplified cells, STEAP3 mediated DMAMCL-induced ferroptosis. This study provides a promising therapeutic target strategy for NB with different *MYCN* expression levels.

## 2. Materials and methods

### 2.1. Cell culture

Five NB cell lines with distinct *MYCN* statuses were used in this study: the *MYCN*-nonamplified SK-*N*-AS (AS) and SH-SY5Y (SY5Y) cell line, *MYCN*-normal NBEB cell line, and the *MYCN*-amplified cell lines NGP and SK-*N*-BE2C (BE2). All cell lines were cultured in RPMI-1640 medium (Solarbio, Beijing, China) supplemented with 10% (v/v) fetal bovine serum and 2 mM glutamine (Bioind, Israel) and maintained at 37 °C in a humidified 5% CO_2_ atmosphere. Cell lines were kindly provided by Dr. Carol J. Thiele of the National Cancer Institute, National Institutes of Health (Bethesda, MD, USA). All cells used in the reported experiments were mycoplasma-negative.

### 2.2. Chemicals and reagents

DMAMCL and MCL were obtained from Nankai University (Tianjin, China). MCL was dissolved in saline to prepare a 20 mM stock solution and MCL was dissolved in DMSO to prepare a 100 mM stock solution. ZnPPIX (2.5  μM, catalog no. MB4231-1) was purchased from Meilunbio Co., Ltd. (Dalian, China). Ferrostatin-1 (Fer-1, 10 μM, catalog no. S7243) was purchased from Selleck Chemicals (Houston, TX, USA). Ciclopirox olamine (CPX, 2.5 μM, catalog no. HY-B0450A) was purchased from MCE (New Jersey, USA). PCR-array kits were purchased from Wc Gene Biotech (catalog no. wc-mRNA0271-H, Shanghai, China). DARTS assay kits ware purchased from Beyotime Biotechnology (catalog no. S3206M, Shanghai, China).

### 2.3. DMAMCL treatment

Based on the IC_50_ concentrations of DMAMCL in each cell line from the previous studies, the working concentrations used in this research were determined [15]. Different NB cell lines were treated with DMAMCL at the following concentrations, respectively: 20 μM for AS cells, 15 μM for NBEB, NGP, and SY5Y cells, and 25 μM for BE2 cells.

### 2.4. Transcriptomic analysis

NGP cells were treated with DMAMCL (15 μM) for 12 hours. The control and DMAMCL-treated groups each containing 2 × 10^7^ cells, and were analyzed in four replicates. Total RNA was isolated using TRIzol reagent (Solarbio, China), and enriched using OligodT magnetic beads (Dynal Biotech, Oslo, Norway). After rRNA depletion, cDNA was generated from the purified mRNA and amplified using PCR. The generated libraries were examined using an Agilent 2100 Bioanalyzer (Agilent Technologies, Santa Clara, CA, USA). Following raw data preprocessing, processed data were obtained through sequential processes of quality filtering, read count quantification, and differential expression analysis. Multiple-comparison corrections for RNA-seq were performed using the ‘Benjamini and Hochberg’ and the ‘Storey and Tibshirani’ methods to adjust *P*-values to adjusted *P*-values. Transcripts that met the threshold criterion (|logFC| > 0.5, adjusted *P* < 0.05) were identified as differentially expressed mRNAs.

### 2.5. Quantification of GSH and MDA levels

Cells were harvested and washed twice with ice-cold phosphate-buffered saline (PBS) via centrifugation at 3000 rpm for 5 minutes. The levels of intracellular reduced GSH and the lipid peroxidation product MDA were quantified using GSH assay kit (catalog no. BC1175; Solarbio, Beijing, China) and MDA assay kit (catalog no. BC0025; Solarbio, Beijing, China), respectively, strictly following the manufacturer's protocols.

### 2.6. Detection of ROS

Intracellular ROS generation was assessed using a fluorescent probe-based ROS Assay Kit (catalog no. S0033S; Beyotime Biotechnology, Shanghai, China). Cells were loaded with 5  μM 2′,7′-dichlorodihydrofluorescein diacetate (DCFH-DA) in serum-free medium and incubated for 20 minutes at 37 °C under light-protected conditions. Following two washes with ice-cold PBS, the fluorescence intensity was quantified using a multi-functional microplate reader with excitation/emission wavelengths set at 488/525 nm or with using fluorescence microscopy. The fluorescence intensity was normalized with distinct baselines: DMAMCL-treated group was normalized to the untreated control (set as 1). For each combination group (a secondary agent + DMAMCL), values were normalized to the corresponding single-agent group (set as 1) to eliminate the intrinsic effect of that agent and isolate the net contribution of DMAMCL in its presence.

### 2.7. Detection of intracellular Fe^2+^

Cells were washed twice with a serum-free medium. FerroOrange (catalog no. F374; Dojindo Laboratories, Kumamoto, Japan) was freshly prepared as a 10 mM stock solution in dimethyl sulfoxide and subsequently diluted to a 10 μM working concentration using serum-free medium. Cells were incubated with FerroOrange working solution (10 μM) for 30 minutes at 37 °C in a humidified 5% CO₂ atmosphere. Then fluorescence images were acquired using fluorescence microscopy, followed by quantitative analysis of the fluorescence intensity using ImageJ software. The fluorescence intensity was normalized to the untreated control of the same cell line, which was set to 1.

### 2.8. Detection of Lipid peroxidation (LPO)

Cells were plated in 35-mm dishes and incubated with 10 μM C11-BODIPY (catalog no. D3861; Thermo Fisher Scientific, Waltham, MA, USA) in HBSS at 37 °C for 30 minutes, and then washed twice with PBS. Fluorescence imaging was performed using a confocal microscope at excitation wavelengths of 590 and 488 nm. Quantitative analysis of fluorescence intensity was subsequently conducted using the ImageJ software, with the ratio of green-to-red fluorescence intensity serving as a quantitative indicator for assessing LPO levels. The values were normalized with distinct baselines: DMAMCL-treated group was normalized to the untreated control (set as 1). For each combination group (a secondary agent + DMAMCL), values were normalized to the corresponding single-agent group (set as 1) to eliminate the intrinsic effect of that agent and isolate the net contribution of DMAMCL in its presence.

### 2.9. Cell viability assay

Cell viability was assessed using a Cell Counting Kit-8 (CCK-8; catalog no. BMU106-CN, Abbkine, Wuhan, Hubei, China). 10 μL CCK-8 reagent was added to each well containing 100 μL culture medium, followed by incubation at 37 °C with 5% CO₂ for 2 hours. Absorbance was measured at 450 nm using a microplate reader.

### 2.10. Live-cell proliferation monitoring

Real-time cell confluence was quantitatively assessed using the Incucyte ZOOM Live-Cell Imaging System (Essen BioScience, Ann Arbor, MI, USA). The cell confluence was used to assess cell proliferation. The percentage of confluence was automatically calculated using the integrated cell confluence module. Endpoint data were presented as Column graphs and were normalized with distinct baselines: the DMAMCL-treated group was normalized to the untreated control (set as 100%). For each combination group (a secondary agent + DMAMCL), values were normalized to the corresponding single-agent group (set as 100%) to eliminate the intrinsic effect of that agent and isolate the net contribution of DMAMCL in its presence. The resulting normalized values for each combination group were then compared to the DMAMCL-alone group.

### 2.11. Quantitative RT-PCR

Cells were harvested for RNA extraction. Total RNA was isolated using the TRIquick Total RNA Extraction Reagent (catalog no. R1100; Solarbio, Beijing, China). Reverse transcription was performed with 5 μg total RNA using the GoScript™ Reverse Transcription System (catalog no. A5003; Promega, Madison, WI, USA). Quantitative real-time PCR analysis was conducted using TB Green™ Premix Ex Taq™ II (catalog no. RR820A; Takara Bio, Shiga, Japan). Primer sequences were listed in Table S1.

### 2.12. Cell transfection

Cells at 60%–70% confluency were transfected with small interfering RNAs or overexpression plasmids. Using the jetPRIME transfection reagent (catalog no. 114-15; Polyplus Transfection, Illkirch-Graffenstaden, France), NB cells were transfected per well with either 5 μL of small interfering RNAs (50 nM) or 2 μg of overexpression plasmids for 14–16 hours. The siRNAs used were: HMOX-siRNA, STEAP3-siRNA, and NRF2-siRNA (Wanze, Anhui, China), and MYCN-siRNA (RIBOBIO, Guangzhou, China). The overexpression plasmids included: MYCN (GeneCopoeia, Guangzhou, China), HMOX1 (Genechem, Shanghai, China), and STEAP3 (Mingsheng, Hefei, China). The validated siRNA target sequences (5′→3′) were as follows: *HMOX1*-siRNA #1: 5′-GCA ACA AAG UGC AAG AUU CTT-3′, 5′-GAA UCU UGC ACU UUG UUG CTT-3′; *HMOX1*-siRNA #2: 5′-GGG UGA UAG AAG AGG CCA ATT-3′, 5′-UUG GCC UCU UCU AUC ACC CTT-3′; *STEAP3*-siRNA: 5′-GAG CAA CCC UAC AGA GCA ATT-3′, 5′-UUG CUC UGU AGG GUU GCU CTT-3′; *NRF2*-siRNA: 5′-GUA AGA AGC CAG AUG UUA A-3′; 5′-UUA ACA UCU GGC UUC UUA C-3′; *MYCN-*siRNA: 5′-CGG AGT TGG TAA AGA ATG A-3′; Control-siRNA: 5′-UUC UCC GAA CGU GUC ACG UTT-3′, 5′-ACG UGA CAC GUU CGG AGA ATT-3′.

### 2.13. Western blotting

Cells were lysed using RIPA lysis buffer (catalog no. R0010; Solarbio, Beijing, China) supplemented with protease/phosphatase inhibitor cocktail. Protein concentrations were determined using a bicinchoninic acid assay kit (catalog no. P0012; Beyotime Biotechnology, Shanghai, China). The protein samples were separated with sodium dodecyl sulfate-polyacrylamide gel electrophoresis, and the protein bands were transferred to PVDF membranes (Millipore, Billerica, MA, USA). Membranes were blocked with 5% skim milk at 20–25 °C. Specific primary antibodies were applied at 4 °C overnight. The following antibodies were used: GPX4 (catalog no. 67763-1-Ig, 1:1000, v/v), HMOX1 (catalog no. 10701-1-AP, 1:1000, v/v), MYCN (catalog no. 10159-2-AP, 1:1000, v/v), KEAP1 (catalog no. 10503-2-AP, 1:1000, v/v), NRF2 (catalog no. 16396-1-AP, 1:1000, v/v), STEAP3 (catalog no. 28478-1-AP, 1:1000, v/v), *β*-tubulin (catalog no. 10068-1-AP, 1:2000, v/v), and GAPDH (catalog no. 10494-1-AP, 1:2000, v/v) from Proteintech Company (Wuhan, China).

### 2.14. Limited proteolysis-coupled mass spectrometry (LIP-MS)

Protein lysates were extracted from NB cells in pre-cooled PBS and quantified by BCA assay. After equal aliquoting, samples were treated with 10 and 30 μM MCL (control with PBS) and incubated at room temperature for 1 hour. Following proteinase K pretreatment, samples were denatured with UA/DOC, reduced with 20 mM DTT at 30 °C for 2 hours, alkylated with 25 mM IAA in the dark for 30 minutes, and diluted with 50 mM NH_4_HCO_3_ to reduce UA/DOC below 1.5 M. Trypsin (2 μg) was added for 16 hours digestion at 37 °C. Peptides were desalted, lyophilized, and reconstituted in 0.1% (v/v) FA before concentration measurement at OD₂₈₀. For DIA analysis, 2 μg peptides spiked with iRT standards were separated on a Vanquish Neo nanoflow system (Thermo Fisher) and analyzed using an Astral mass spectrometer. Data were processed in Spectronaut with a Q value cutoff of 0.01.

### 2.15. Molecular docking and Molecular dynamics (MDs)

Obtain the 3D structure of MCL from the PubChem database, and retrieve the 3D structure of KEAP1 Kelch domain was obtained from the PDB database (PDB ID: 6TYM). Prior to docking, the MCL ligand was subjected to hydrogen addition and energy minimization, while the KEAP1 receptor was processed by adding hydrogen atoms, removing water molecules, and deleting the original ligand. Subsequently, semi-flexible molecular docking was performed using AutoDock-GPU. The docking grid box center coordinates (Å) were set to x = −5.113, y = −22.867, z = −20.075, and the grid box dimensions (Å) were set to x = 94, y = 93, z = 81. MDs were performed using GROMACS 2024.3. Receptor topology was generated with pdb2gmx using the AMBER99SB-ILDN force field, while ligand topology was prepared with acpype and the GAFF2 force field. The system was solvated in a cubic water box with a 1.2 nm solvent layer and neutralized with ions. Simulations included energy minimization, followed by equilibration and production runs in the NPT ensemble. Key parameters: Verlet cut-off scheme, short-range electrostatics and van der Waals cut-off at 1.0 nm, long-range electrostatics via PME, bond constraints with LINCS, temperature coupling at 310 K (Nose-Hoover), pressure coupling at 1 bar (Parrinello-Rahman), integration step of 2 fs, and total duration of 500 ns. Trajectory analysis involved calculating RMSD, Rg, distance, Gibbs free energy, and energy profiles using GROMACS and dit tools.

### 2.16. Drug affinity responsive target stability (DARTS)

Proteins were extracted by lysing cells with lysis buffer. The samples were centrifuged at 4 °C and 12,000 × g for 5 min. The supernatant was collected, and protein concentration was quantified using the BCA assay. The proteins were then incubated with 25 μM MCL or PBS at room temperature for 1 h. Subsequently, Protease was added at a ratio of 1:2000 (v/v, protease to total protein) and incubated at 40 °C for 30 minutes. The reaction was immediately stopped by adding 1 μL of Protease Inhibitor Cocktail (100×). Finally, the proteins were denatured and subjected to Western blot.

### 2.17. Cell thermal shift assay (CETSA)

Proteins were extracted by lysing cells with RIPA buffer. The extracted proteins were incubated with 25 μM MCL or PBS at room temperature for 1 hour. Subsequently, the protein samples were heated separately at 45, 50, 55, and 60 °C for 3 minutes. After heating, the samples were centrifuged at 4 °C and 12,000 × g for 20 minutes. The supernatant was then collected for protein denaturation and analyzed by Western blot.

### 2.18. Immunofluorescent staining

Cells were fixed with 4% paraformaldehyde and permeabilized with 0.2% Triton X-100. After blocking with 5% goat serum for 1 hour, cells were incubated with NRF2 antibody (catalog no. 16396-1-AP, 1:200, v/v, Proteintech, Wuhan, China) for 1 hour. Then cells were incubated with fluorescently labeled secondary antibody (ab150113, 1:1000, v/v, Abcam, Cambridge, UK) for 1 hour. After washing three times, the visualization of fluorescence signals was acquired though the confocal laser scanning microscope.

### 2.19. Electroporation

Nucleofector working solution was prepared by mixing Cell line Nucleofector solution and Supplement at a ratio of 81.8 μL:18.2 μL per transfection. Subsequently, 3 μg of shRNA or overexpression plasmids were added to each mixture. Cells were resuspended in the prepared working solution at a density of 1 × 10^7^ cells per transfection. Transfection was performed using the Lonza Nucleofector system under program ‘A023’.

### 2.20. In vivo study

Female BALB/c nude mice (4–6 weeks old) were purchased from Beijing Huafukang Bioscience Co., Ltd. All mice were housed under specific pathogen-free (SPF) conditions with free access to standard chow and water. The animal room was maintained at 20–24 °C with a 12-hour light/dark cycle. The mice were randomly assigned into groups. NB cells (AS: 2 × 10^6^, NGP: 3 × 10^6^, BE2: 2 × 10^6^) were suspended in 100 μL of 1:1 mixture of PBS/Matrigel and subcutaneously injected into the right flank. This study involved three independent animal experiments. In Experiment 1, AS and NGP xenograft mice were divided into Control and DMAMCL groups (*n* = 4 per group). In Experiment 2, BE2 xenograft mice were assigned to six groups: Control, sh-HMOX1, DMAMCL, sh-HMOX1 + DMAMCL, Empty vector, and OE-HMOX1. One non-tumor-bearing mouse was excluded from the sh-HMOX1 + DMAMCL group, resulting in a final *n* = 9 for this group, while all other groups remained at *n* = 10. In Experiment 3, AS xenograft mice were divided into Control, sh-STEAP3, DMAMCL, sh-STEAP3 + DMAMCL, Empty vector, and OE-STEAP3 groups, each group with *n* = 10. When tumor volumes reached 100–150 mm^3^, the following treatments were administered: In Experiment 1, mice received either DMAMCL (100 mg/kg) or saline placebo via daily oral gavage for 10 days, then tumor tissues were collected for the detection of Fe levels, MDA, and mitochondrial morphology. In Experiments 2 and 3, the Control, sh-HMOX1, DMAMCL, and sh-HMOX1 + DMAMCL groups in the BE2 xenograft model, and the Control, sh-STEAP3, DMAMCL, and sh-STEAP3 + DMAMCL groups in the AS xenograft model—were treated daily via gavage with DMAMCL (100 mg/kg) or saline. The remaining groups (Empty vector and OE-HMOX1/OE-STEAP3) received no treatment. The dose of DMAMCL was selected with reference to that employed in a prior study [15]. The cage locations and the sample processing order were randomized to minimize potential confounding factors. Tumor size was measured, and survival was monitored. The mice were euthanized by CO_2_ inhalation when the tumor diameter approached 15 mm. The experimental protocols involving animals were conducted in accordance with ethical standards and received approval from the Experimental Animal Ethical Committee of Shengjing Hospital of China Medical University (Approval No. 2022PS352K; Approval No. 2025PS1281K).

### 2.21. Statistical analysis

Data are presented as mean ± SD or mean ± SEM. All quantitative data included at least three independent biological replicates per group or independent experiment. For data with n ≥ 10, the Shapiro–Wilk test was used to assess normality. Differences between two groups with normality were analyzed using the Student's t-test, comparisons between two groups without normality were analyzed through Wilcoxon signed-rank test. Differences among three or more groups were evaluated with one-way or two-way ANOVA, followed by Tukey's HSD test for post-hoc multiple comparisons. Survival curves were compared using the Log-rank test. Statistical analyzes were performed using GraphPad Prism 8.0. *P* < 0.05 was considered statistically significant.

## 3. Results

### 3.1. RNA sequencing analysis of DMAMCL-treated NGP cells

**Figure 1.**
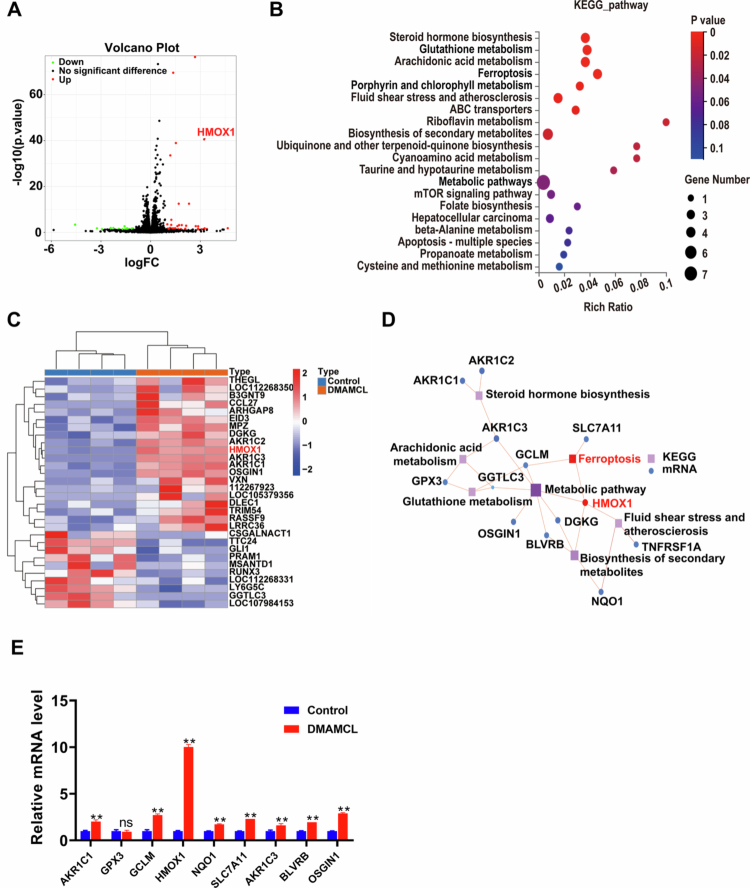
RNA sequencing analysis of DMAMCL-treated NGP cells. (A) NGP cells were treated with 15  μM DMAMCL for 12 hours followed by total RNA isolation for transcriptome sequencing. Differentially expressed genes (DEGs) were visualized in a volcano plot. (B) Significantly enriched KEGG pathways of the DEGs. In the network diagram, rectangular and circular nodes correspond to KEGG pathways and mRNA entities, respectively. (C) The heatmap visualization of DEGs displayed sample clusters along the horizontal axis and gene clusters along the vertical axis (*n* = 4 technical replicates). (D) Network diagram of the interactions among the DEGs and the significantly enriched KEGG pathways, circular nodes represent genes and square-shaped nodes denoted biological pathways. (E) RT–PCR was performed to validate the expression levels of 11 DEGs identified through transcriptome analysis, *n* = 3 independent biological replicates. Data were represented as mean ± SD. **P < 0.01, non-significant (ns).

To identify the potential antitumor mechanisms of DMAMCL in NB, RNA-seq was performed in NGP cells. Using the threshold of |log₂FC| > 0.5 and adjusted *P* < 0.05, a total of 137 differentially expressed genes (DEGs) were identified in DMAMCL-treated cells compared to the controls, as visualized in the volcano plot (Figure 1A). Kyoto Encyclopedia of Genes and Genomes (KEGG) pathway analysis indicated that the top 5 pathways enriched with DEGs were steroid hormone biosynthesis, glutathione metabolism, arachidonic acid metabolism, ferroptosis, and porphyrin and chlorophyll metabolism, all of which are closely associated with ferroptosis (Figure 1B). Hierarchical cluster analysis was performed to visualize the differential expression of the top 20 upregulated and top 10 downregulated DEGs in all samples (Figure 1C). The KEGG pathways of the DEGs were ranked by the number of genes in the pathway, and the top 10 pathways with the most significant numbers of genes are displayed (Figure 1D). Based on the results in Figure 1C and D, we selected 11 differentially expressed genes closely associated with ferroptosis and its related pathways for validation at the mRNA level, to identify key genes that may mediate DMAMCL-induced ferroptosis. Among the selected genes, the mRNA level of *HMOX1* was significantly elevated by DMAMCL (Figure 1E). These results suggested that ferroptosis served as a critical pathway underlying the antitumor effects of DMAMCL, with HMOX1 potentially acting as a pivotal factor mediating ferroptosis in NB cells.

### 3.2. DMAMCL induces ferroptosis *in vitro* and *in vivo* in NB

**Figure 2.**
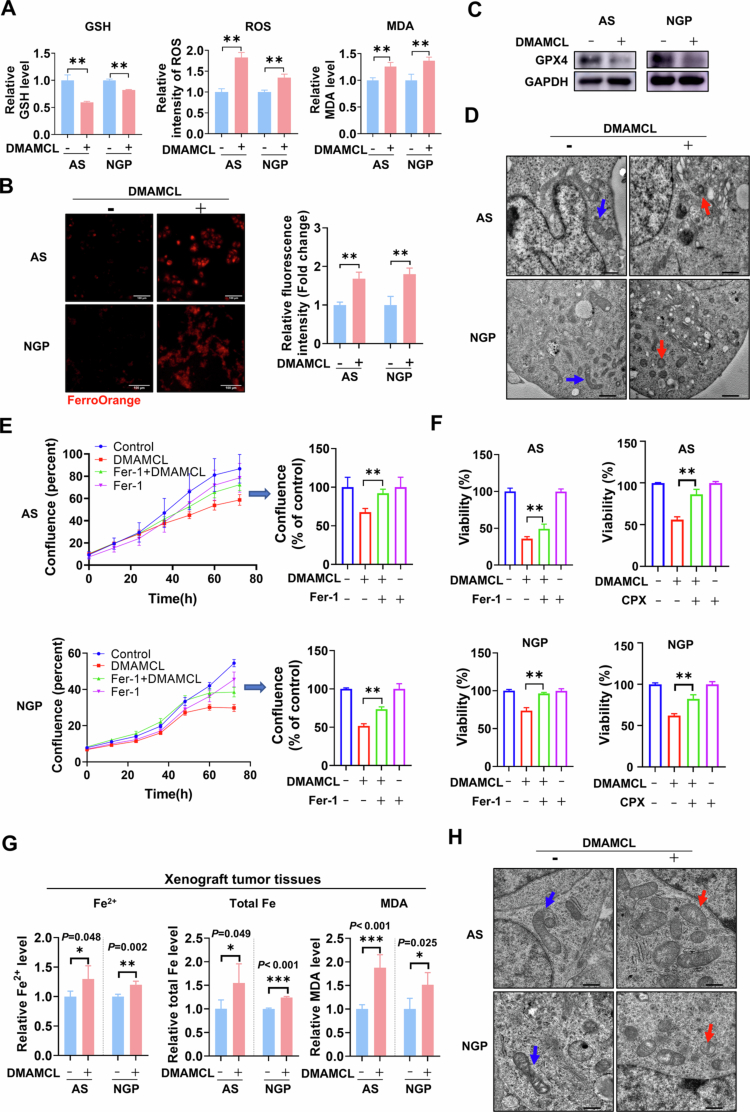
DMAMCL treatment induces ferroptosis *in vitro* and *in vivo* in NB. (A) The GSH, ROS, and MDA were quantitatively measured in AS and NGP cell lines following 24-hour exposure to DMAMCL (20 μM for AS cells, 15 μM for NGP cells). (B) The fluorescence intensity of Fe^2+^ (red) was detected with FerroOrange. Quantitative analysis of fluorescence intensity was performed using ImageJ software. scale bar, 100 μm. (C) Protein expression levels of GPX4 in cells treated with DMAMCL for 24 hours. (D) Morphological changes in mitochondria were observed with electron microscopy. Blue arrows indicate normal mitochondria, and red arrows indicate abnormal mitochondria. Scale bar, 1 μm. (E) Analysis of cell confluence of AS and NGP cells treated with DMAMCL for 48 hours in the presence or absence of Fer-1. Cell confluence was observed using the IncuCyte ZOOM live cell imaging system and the data were presented as line graph. Endpoint data were presented as Column graphs and were normalized with distinct baselines: DMAMCL-treated group was normalized to the control (set as 100%), Fer-1 + DMAMCL group was normalized to Fer-1 group (10 μM). (F) Analysis of cell viability of AS and NGP cells treated with DMAMCL for 48 hours in the presence or absence of Fer-1 and CPX. (G) The xenograft tumors in mice were treated with DMAMCL (100 mg/kg) daily for 10 consecutive days. Then xenograft tumor tissues were harvested for Fe^2+^, total iron, and MDA levels detection mice. (*n* = 4 mice). (H) Morphological changes of mitochondria in xenograft tumor tissues were observed with electron microscopy. Blue arrows indicate normal mitochondria, and red arrows indicate abnormal mitochondria. Scale bar, 500 nm. Data were represented as mean ± SD, *n* ≥ 3 independent biological replicates. *P < 0.05, **P < 0.01, ***P < 0.001.

To further identify whether DMAMCL induced ferroptosis in NB cells, AS and NGP cells were treated with DMAMCL for 24 hours, and then ferroptosis-associated biomarkers were detected. The results showed that DMAMCL significantly decreased GSH levels and increased ROS, MDA, Fe^2+^, and LPO levels (Figure 2A and B and Fig S1). GPX4 is one of the key indicators for assessing ferroptosis, and we measured its expression level after DMAMCL treatment in NB cells. The results showed that DMAMCL decreased the protein expression level of GPX4 (Figure 2C). Furthermore, as shown in Figure 2D (red arrows), mitochondria appeared to shrink and mitochondrial cristae broke up after DMAMCL treatment. Additionally, the ferroptosis inhibitor Fer-1 and CPX significantly blocked DMAMCL-induced cell death (Figure 2E and F).

To investigate whether DMAMCL induced ferroptosis in *vivo*, AS and NGP tumor-bearing mice were treated with DMAMCL (100 mg/kg) daily for 10 days. Fresh tumor tissues were collected to detect Fe^2+^, total iron, and MDA levels. The results showed that levels of Fe^2+^, total iron, and MDA in the DMAMCL treatment group were significantly higher than those in the control group (Figure 2G). Ultrastructural analysis of tumor tissues using electron microscopy revealed significant mitochondrial alterations, including volumetric reduction and cristae fragmentation (red arrows) (Figure 2H). These results suggested that DMAMCL induced ferroptosis in NB both *in vitro* and *in vivo*.

### 3.3. Knockdown of *HMOX1* exerted cell type-specific effects on the effects of DMAMCL

**Figure 3.**
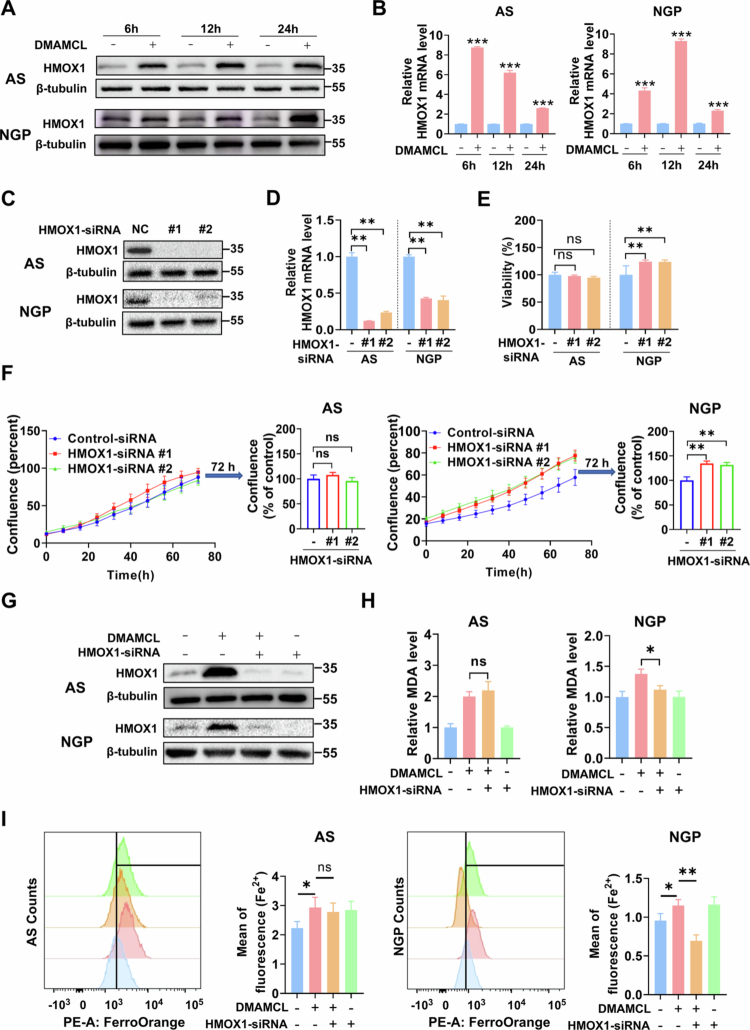
Knockdown of HMOX1 exerted cell type-specific effects on the effects of DMAMCL. (A, B) The expression of *HMOX1* at protein (A) and mRNA (B) levels in AS and NGP cells was detected after DMAMCL treatment for 6, 12, and 24 hours. (C-D) The expression of *HMOX1* at protein and mRNA levels transfected with *HMOX1-*siRNAs or control-siRNA. (E, F) Cell viability and cell confluence of AS and NGP cells after transfecting with *HMOX1-*siRNAs or control-siRNA. (G) HMOX1 protein levels in AS and NGP cells transfected with *HMOX1*-siRNA or control-siRNA, followed by DMAMCL treatment, were detected by Western blotting. (H) MDA levels in AS and NGP cells transfected with control-siRNA or *HMOX1-*siRNA followed by 24 hours DMAMCL treatment were quantified. (I) Fe^2+^ levels of AS and NGP cells after transfecting with *HMOX1*-siRNA or control-siRNA and followed by DMAMCL treatment for 24 hours were detected using FerroOrange and quantitatively analyzed by flow cytometry. Data were represented as mean ± SD, *n* ≥ 3 independent biological replicates. *P < 0.05, **P < 0.01, non-significant (ns).

Transcriptome sequencing and real-time PCR analyzes showed that the expression level of *HMOX1* was significantly increased after DMAMCL treatment (Figure 1A, C, and E). To further identified whether HMOX1 mediated DMAMCL-induced ferroptosis, we first detected the expression level of *HMOX1* using Western blotting and RT-qPCR after DMAMCL treatment for 6  h, 12  h, and 24  h in AS and NGP cells. The results showed that DMAMCL significantly upregulated the *HMOX1* expression levels (Figure 3A and B). Additionally, we knocked down *HMOX1* using siRNA in AS and NGP cells (Figure 3C and D), and detected cell confluence and cell viability. The results showed that knockdown of *HMOX1* did not affect cell viability or cell confluence in AS cells, but increased cell viability and cell confluence in NGP cells (Figure 3E and F). Since both *HMOX1*-siRNA #1 and #2 exhibited similar effects in AS and NGP cells, *HMOX1*-siRNA #1, which exhibited the optimal knockdown efficiency, was selected for subsequent experiments. Western blotting results showed that *HMOX1* knockdown blocked DMAMCL-induced upregulation of *HMOX1* in both AS and NGP cells (Figure 3G). Additionally, we detected MDA, Fe^2+^, and ROS levels. The results demonstrated that *HMOX1* knockdown attenuated DMAMCL-induced increases in MDA, Fe^2+^, and ROS levels only in NGP cells, with no significant effects observed in AS cells (Figure 3H, I and Figure S2A). These results suggested that knockdown of *HMOX1* exerted cell type-specific effects on the effects of DMAMCL.

### 3.4. HMOX1 mediated DMAMCL-induced ferroptosis in *MYCN*-amplified NB cells but not in *MYCN*-nonamplified AS cells

**Figure 4.**
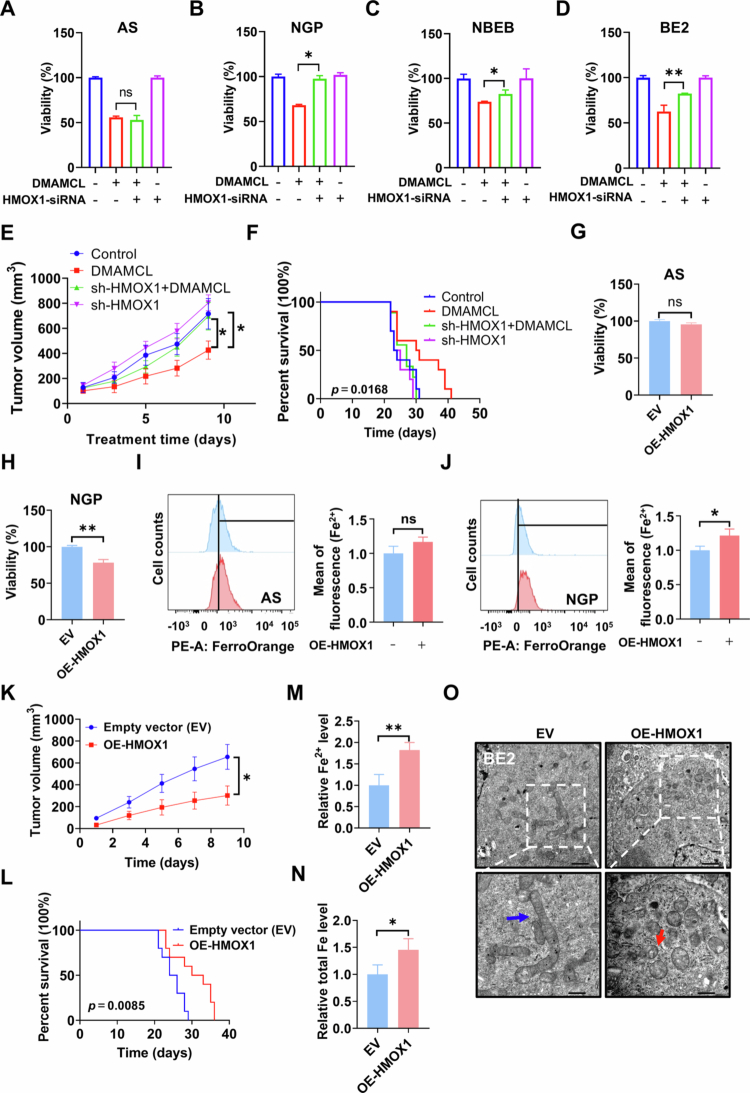
HMOX1 mediated DMAMCL-induced ferroptosis in *MYCN*-amplified NB cells but not in *MYCN-*nonamplified AS cells. (A–D) Cell viability of AS, NGP, NBEB, and BE2 cells transfected with *HMOX1-*siRNA or control-siRNA and followed by DMAMCL treatment. (E) Tumor growth curves in BE2 xenograft mice following 9-day sustained administration of placebo or DMAMCL (100 mg/kg), sh-HMOX1 + DMAMCL (*n* = 9 mice), other groups (*n* = 10 mice), data represent the mean ± SEM. (F) Survival curves of BE2 xenograft mice treated with placebo or DMAMCL (100 mg/kg), sh-HMOX1 + DMAMCL (*n* = 9 mice), other groups (*n* = 10 mice). (G–H) Cell viability was assessed using CCK-8 assays after overexpressing *HMOX1* for 72 h in AS and NGP cells. (I–J) Fe^2+^ levels of AS and NGP cells after overexpressing *HMOX1* were detected using FerroOrange and quantitatively analyzed by flow cytometry. (K) Tumor growth curve in BE2 xenograft mice, *n* = 10 mice per group, data represent the mean ± SEM. (L) Survival curves of BE2 xenograft mice. (*n* = 10 mice per group, as analyzed by Log-rank test). (M) Measurement of Fe^2+^ levels in BE2 xenograft tumor tissues using an iron ion assay kit. (N) Measurement of total Fe levels in BE2 xenograft tumor tissues using an iron ion assay kit. (O) Morphological changes of mitochondria in BE2 xenograft tumor tissues were observed with electron microscopy. Blue arrows indicate normal mitochondria, and red arrows indicate abnormal mitochondria. Scale bar, 500 nm. Data were represented as mean ± SD or mean ± SEM, *n* ≥ 3 independent biological replicates, *P < 0.05, **P < 0.01, non-significant (ns).

Given that *HMOX1* knockdown exerted cell type-specific effects on the effects of DMAMCL, we included two additional cell lines, *MYCN*-normal NBEB cells and *MYCN*-amplified BE2 cells for the following studies. We suppressed HMOX1 in AS, NGP, NBEB, and BE2 cells, treated them with DMAMCL, and subsequently measured cell viability and confluence. Similar to the results in AS and NGP cells, *HMOX1* knockdown in both BE2 and NBEB cells abolished the upregulation of HMOX1 induced by DMAMCL (Figure S2B). We also observed that *HMOX1* knockdown attenuated DMAMCL-induced decreases in cell viability and confluence in NGP, NBEB, and BE2 cells, whereas it had no effects on the cell confluence and viability of AS cells (Figure 4A–D and Figure S3A-D). Additionally, the HMOX1 inhibitor Zinc Protoporphyrin (ZnPPIX) attenuated the DMAMCL-induced cell death in NGP, NBEB, and BE2 cells, but not in AS cells (Figure S3E). Additionally, we established *MYCN*-amplified BE2 xenograft tumor models. Following tumor establishment, daily administration of placebo or DMAMCL (100 mg/kg) was initiated. Tumor growth curve results showed that *HMOX1* knockdown abrogated DMAMCL-induced tumor growth suppression (Figure 4E and Figure S4). Survival analysis demonstrated that significantly reduced survival time in the DMAMCL-treated sh-*HMOX1* group compared to the DMAMCL group (Figure 4F).

Given that HMOX1 was upregulated by DMAMCL, we investigated the effect of HMOX1 by transfecting overexpression plasmids and then measured cell viability and intracellular Fe^2+^ levels. The results demonstrated that *HMOX1* overexpression did not affect cell viability in *MYCN*-nonamplified AS cells, whereas it significantly suppressed viability in *MYCN*-amplified NGP and BE2, and *MYCN*-normal NBEB cell lines (Figure 4G–H and Figure S5A). Meanwhile, *HMOX1* overexpression significantly increased intracellular Fe^2+^ levels in NGP, BE2, and NBEB cells, but not in AS cells (Figure 4I–J and Figure S5B, C). Additionally, we established a BE2 xenograft model with *HMOX1* overexpression in nude mice. Tumor growth curves showed that overexpression of *HMOX1* significantly suppressed tumor growth compared to the empty vector (EV) group (Figure 4K and Figure S5D). Furthermore, survival analysis demonstrated a significant prolongation of survival in the *HMOX1*-overexpressing group (Figure 4L). Consistent with the increased intracellular Fe^2+^, the tumor tissues in the *HMOX1*-overexpressing group exhibited a significant accumulation of both Fe^2+^ and total iron (Figure 4M, N). Furthermore, the mitochondria within these tumors showed a marked reduction in size and displayed cristae disruption (Figure 4O). Additionally, we used a 498-sample NB dataset (GSE49710) of the R2 database to assess the prognostic survival analysis, which showed *HMOX1* expression was significantly associated with prognosis. Patients with NB and higher HMOX1 expression demonstrated a longer survival time than those with lower expression (Figure S5E). Together, these results demonstrate that HMOX1 mediated DMAMCL-induced ferroptosis in *MYCN*-amplified NB cells but not in *MYCN*-nonamplified AS cells.

### 3.5. HMOX1-mediated DMAMCL-induced ferroptosis exhibits MYCN-associated susceptibility

**Figure 5.**
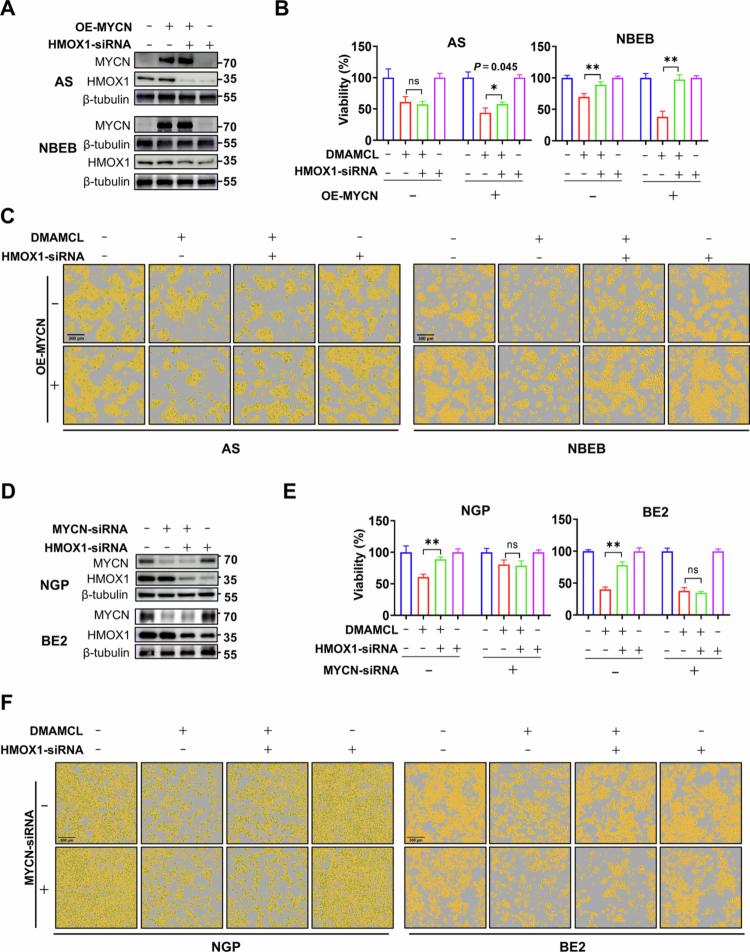
HMOX1-mediated DMAMCL-induced cell death exhibits MYCN-associated susceptibility. (A) HMOX1 and MYCN protein expression levels in AS and NBEB following transfection with either *MYCN*-overexpressing plasmid or empty vector, in combination with *HMOX1*-siRNA or control-siRNA. (B) Cell viability was analyzed between *HMOX1-*siRNA + DMAMCL group and the DMAMCL-alone treatment group in AS and NBEB cells transfected with or without *MYCN*-overexpressing plasmid. (C) Representative images of cells transfected with *HMOX1-*siRNA or control-siRNA followed by DMAMCL treatment in AS and NBEB cells transfected with or without *MYCN*-overexpressing plasmid. Scale bar, 300 μm. (D) HMOX1 and MYCN protein expression levels in NGP and BE2 following transfection with *MYCN*-siRNA or control-siRNA, in combination with *HMOX1-*siRNA or control-siRNA. (E) Cell viability was analyzed between *HMOX1*-siRNA + DMAMCL group and the DMAMCL-alone treatment group in NGP and BE2 cells transfected with or without *MYCN*-siRNA. (F) Representative images of cells transfected with *HMOX1-*siRNA or control-siRNA followed by DMAMCL treatment in NGP and BE2 cells transfected with or without *MYCN*-siRNA. Scale bar, 300 μm. Data were represented as mean ± SD, *n* = 3 independent biological replicates. *P < 0.05, **P < 0.01, non-significant (ns).

We demonstrated that HMOX1-mediated DMAMCL-induced ferroptosis exhibited cell-type differences. To investigate whether the effect of *HMOX1* upregulation in DMAMCL-induced ferroptosis is MYCN-dependent, after overexpressing *MYCN* and knocking down *HMOX1* in AS and NBEB cells via plasmid and siRNA transfection, respectively, the cells were treated with DMAMCL. Western blotting results demonstrated that neither *MYCN* overexpression affected *HMOX1* expression, nor *HMOX1* knockdown affected *MYCN* expression (Figure 5A), indicating that there was no direct expression regulation between *MYCN* and *HMOX1*. For the changes of cell viability, we found that *MYCN* overexpression enabled *HMOX1* knockdown to significantly block the DMAMCL-induced cell death in AS cells, although *HMOX1* knockdown did not affect DMAMCL-induced cell death under MYCN-deficient conditions (Figure 5B, left panel). The cell confluence changes showed a trend consistence with the cell viability (Figure S6A). In *MYCN*-normal NBEB cells we found that *HMOX1* knockdown resulted in a significantly stronger blockage of DMAMCL-induced cell death when *MYCN* was overexpressed compared to that under basal *MYCN* expression conditions (59.3% vs. 19.1%) (Figure 5B, right panel). Similar changes were observed in cell confluence (33.6% vs. 19.6%) (Figure S6B). The changes under microscopy of AS and NBEB cells in different conditions showed a trend consistence with the results of cell viability and cell confluence (Figure 5C).

Additionally, we knocked down *MYCN* and *HMOX1* by transfecting *MYCN*-siRNA and *HMOX1*-siRNA in *MYCN*-amplified NGP and BE2 cells, respectively, and then treated these cells with DMAMCL to detect the *MYCN* and *HMOX1* expression levels, cell viability and cell confluence. Western blotting results showed that there was no mutual regulation between *MYCN* and *HMOX1* (Figure 5D). Cell viability results showed that after *MYCN* knockdown, *HMOX1* knockdown failed to block DMAMCL-induced cell death in NGP and BE2 cells, compared to a significant blocking effect under conditions of high MYCN levels (Figure 5E). The cell changes under microscopy and cell confluence showed a trend consistent with the cell viability (Figure 5F and Figure S6C, D). These results indicated that HMOX1-mediated DMAMCL-induced ferroptosis exhibited MYCN-associated susceptibility.

### 3.6. DMAMCL binding to KEAP1 promoted NRF2/HMOX1 activation in*MYCN*-amplified NB cells

**Figure 6.**
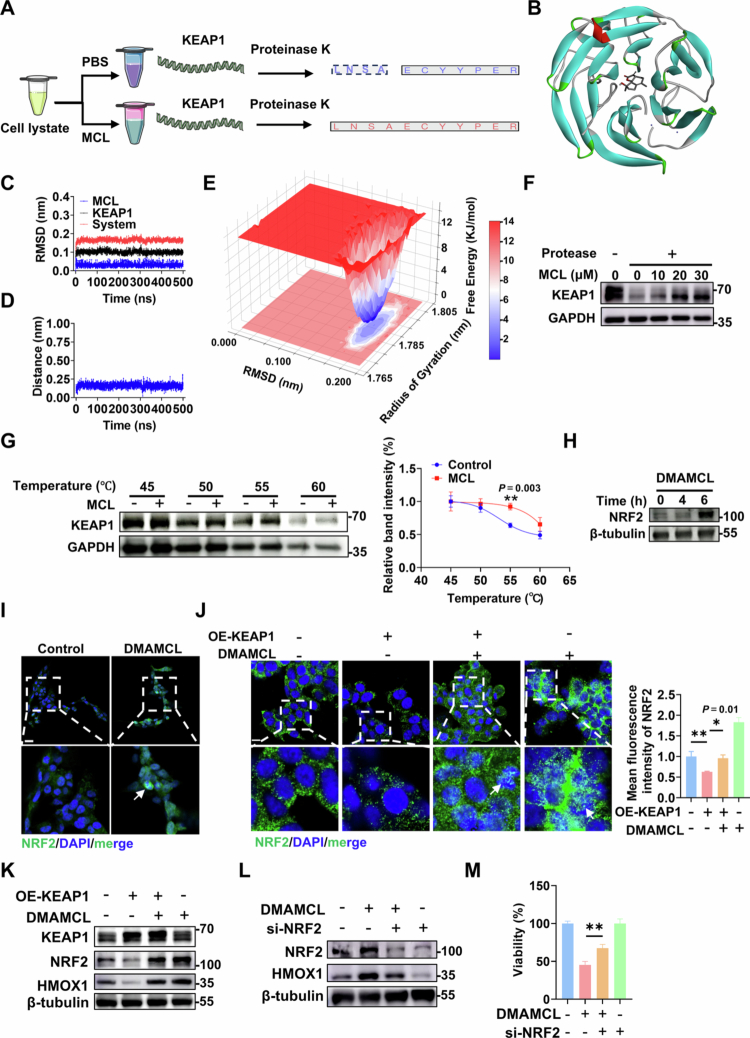
DMAMCL binding to KEAP1 promoted NRF2/HMOX1 activation in *MYCN*-amplified NB cells. (A) Identification of the direct binding targets of MCL by LIP-MS. (B) Molecular docking result of MCL and KEAP1. (C) RMSD results of MCL and KEAP1. (D) Analysis of the distance change between MCL and KEAP1 from the molecular dynamics simulation results. (E) Free energy landscape of MCL binding to KEAP1. (F) Lysates from BE2 cells were incubated with increasing concentrations of DMAMCL (0, 10, 20, 30 µM) and then digested with protease. GPX4 protein levels were detected by Western blotting. (G) Left panel, the thermal stability of KEAP1 was detected by CETSA; Right panel, the KEAP1 bands were quantified, and the thermal stability curve was plotted. (H) Western Blot analysis detected the expression levels of NRF2 in cells treated with DMAMCL (25  μM) for 4 and 6 hours. (I) Immunofluorescence was used to detect the localization of NRF2 in cells following DMAMCL treatment for 6 hours in BE2 cells. White arrows indicate nuclear localization of the protein. Scale bar, 50 μm. (J) After overexpressing KEAP1, the cells were treated with DMAMCL for 6 hours, immunofluorescence was used to detect the expression levels and localization of NRF2 in BE2 cells. White arrows indicate nuclear localization of the protein. (K) After overexpressing KEAP1 in BE2 cells, the cells were treated with DMAMCL for 6 hours, Western blotting was used to detect the expression levels of KEAP1, NRF2, and HMOX1. (L) NRF2‑knockdown BE2 cells were treated with DMAMCL for 6  hours, and the protein expression levels of NRF2 and HMOX1 were examined by Western blotting. (M) NRF2‑knockdown BE2 cells were treated with DMAMCL for 48 hours, and cell viability were examined CCK-8. Data were represented as mean ± SD, *n* = 3 independent biological replicates. *P < 0.05, **P < 0.01.

To further identify the mechanisms by which DMAMCL upregulates HMOX1 in NB cells, we incubated MCL, the active form of DMAMCL, with total protein extracts from NB cells, and subsequently employed limited proteolysis‒mass spectrometry (LiP‒MS) to identify the direct binding targets of MCL. We identified differential peptide fragments of KEAP1, suggesting a potential interaction between MCL and KEAP1 (Figure 6A). It has been reported that KEAP1 suppresses NRF2, a transcription factor that regulates the expression of HMOX1 [28]. Given our finding that DMAMCL significantly upregulates HMOX1, we further investigated whether DMAMCL regulates the NRF2/HMOX1 axis by binding to KEAP1. NRF2 binds to the Kelch domain of KEAP1. Therefore, competitive displacement of NRF2 by MCL would require MCL to bind the same domain. To investigate this, we performed molecular docking and molecular dynamics simulations. The docking results showed that MCL binds to the Kelch domain of KEAP1 (Figure 6B). The root-mean-square deviation (RMSD) analysis indicated that the MCL-KEAP1 complex achieved conformational convergence and high structural stability and the distance between MCL and KEAP1 remained stable, indicating a stable binding interaction (Figure 6C–D). Free energy landscape (FEL) analysis identified the most thermodynamically stable conformation of the MCL-KEAP1 complex, which corresponds to the global free energy minimum (Figure 6E). To validate the above predicated results, we performed DARTS and CETSA experiments, which demonstrated that MCL directly binds to KEAP1 (Figure 6F–G).

We next treated BE2 cells with DMAMCL, and the expression levels and subcellular localization of NRF2 were detected. We found that DMAMCL significantly increased the expression levels of NRF2, and induced the accumulation of NRF2 in the nucleus (Figure 6H and I). Overexpression of KEAP1 significantly downregulated the expression levels of both NRF2 and HMOX1. This downregulation was rescued by DMAMCL treatment, which increased the nuclear accumulation of NRF2 (Figure 6J and K).

Furthermore, we knocked down NRF2 to investigate whether it is involved in regulating HMOX1-mediated ferroptosis induced by DMAMCL. The results showed that NRF2 knockdown effectively blocked the upregulation of HMOX1 expression upon DMAMCL treatment and significantly attenuated DMAMCL-induced cell death (Figure 6L and M). In summary, these results suggested that in *MYCN*-amplified NB cells, DMAMCL bound to KEAP1, leading to increased NRF2 levels, which upregulated HMOX1 and ultimately induced ferroptosis.

### 3.7. STEAP3 mediated DMAMCL-induced ferroptosis in *MYCN*-nonamplified NB cells

**Figure 7.**
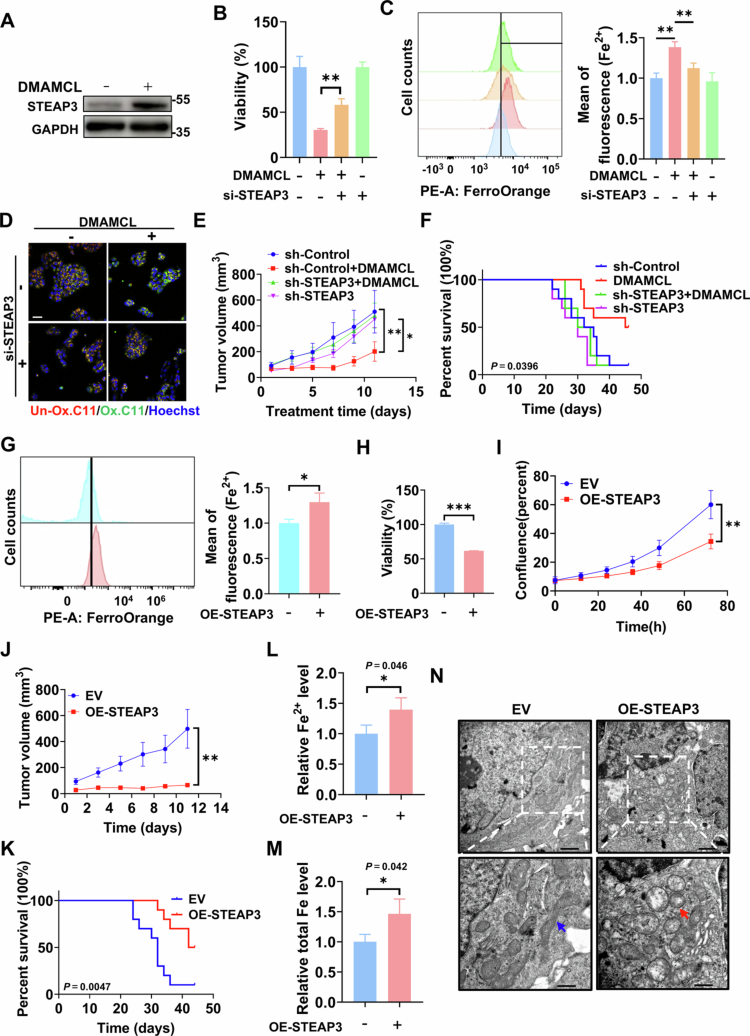
STEAP3 mediated DMAMCL-induced ferroptosis in *MYCN*-nonamplified NB cells. (A) Western blotting detected STEAP3 protein levels after DMAMCL treatment for 6 hours in AS cells. (B) Cell viability of AS after transfecting with *STEAP3*-siRNA or control-siRNA and followed by DMAMCL treatment. (C) Fe^2+^ levels of AS after transfecting with *STEAP3*-siRNA or control-siRNA and followed by DMAMCL treatment for 24 hours were detected using FerroOrange and quantitatively analyzed by flow cytometry. (D) LPO levels of AS after transfecting with *STEAP3*-siRNA or control-siRNA and followed by DMAMCL treatment for 8 hours were detected using BODIPY-C11 and observed using confocal microscope, scale bar, 50 μm. (E) Tumor growth curves in AS xenograft mice following 11-day sustained administration of placebo or DMAMCL (100 mg/kg), *n* = 10 mice per group, data were represented as the mean ± SEM. (F) Survival curves of AS xenograft mice treated with placebo or DMAMCL (100  mg/kg), *n* = 10 mice per group. (G) Fe^2+^ levels of AS after overexpressing *STEAP3* were detected using FerroOrange and quantitatively analyzed by flow cytometry. (H) Cell viability of AS after overexpressing *STEAP3* was detected using CCK-8 assays. (I) Cell confluence of AS after overexpressing *STEAP3* was detected using IncuCyte ZOOM live cell imaging system. (J) Tumor growth curve of AS xenograft mice, *n* = 10 mice per group, data were represented as the mean ± SEM. (K) Survival curves of AS xenograft mice, *n* = 10 mice per group. (L) Measurement of Fe^2+^ levels in AS xenograft tumor tissues using an iron ion assay kit. (M) Total iron levels in AS xenograft tumor tissues were measured using an iron ion assay kit. (N) The mitochondrial morphology in AS xenograft tumor tissues was observed using a transmission electron microscope. Blue arrows indicate normal mitochondria, and red arrows indicate abnormal mitochondria. Data were represented as mean ± SD or mean ± SEM, *n* ≥ 3 independent biological replicates, *P < 0.05, **P < 0.01, ***P < 0.001.

Since *HMOX1* upregulation did not mediate DMAMCL-induced iron accumulation and ferroptosis in AS cells (Figure 3I and 4A), we sought to identify key genes mediating DMAMCL-induced ferroptosis in *MYCN*-nonamplified NB cells. To this end, we used a ferroptosis PCR array to detect the expression of iron metabolism-associated genes after DMAMCL treatment (Figure S7A). We validated the protein expression of these genes by Western blot. The results showed that the expression of *STEAP3* was upregulated by DMAMCL in AS cells (Figure 7A). We then detected cell viability, intracellular Fe^2+^ and LPO levels after *STEAP3* knockdown followed by DMAMCL treatment in *MYCN*-nonamplified NB cells. The results showed that *STEAP3* knockdown significantly attenuated DMAMCL-induced cell death, intracellular Fe^2+^ elevation, and LPO level elevation in AS and SY5Y cells (Figure 7B–D and Figure S7B-F). Additionally, we established a subcutaneous xenograft mouse model using AS cells. Tumor growth curve results revealed that *STEAP3* knockdown abrogated DMAMCL-induced tumor growth suppression (Figure 7E and Figure S7G). Survival analysis demonstrated significantly reduced survival in the DMAMCL-treated sh-STEAP3 group compared to the DMAMCL group (Figure 7F).

To further investigate the effects of STEAP3 in *MYCN*-nonamplified NB cells, we overexpressed *STEAP3* using overexpression plasmids and then detected the levels of Fe^2+^, cell viability and confluence. The results showed that overexpression of *STEAP3* significantly increased the levels of Fe^2+^ in AS and SY5Y cells and decreased cell viability and confluence (Figure 7G–I and Figure S8A-D). Furthermore, both ferroptosis inhibitors Fer-1 and CPX significantly rescued the decrease in cell viability induced by STEAP3 overexpression (Figure S8E). *In vivo* studies, overexpression of *STEAP3* significantly inhibited tumor growth and prolonged the survival of mice compared to the EV group (Figure 7J and K and Figure S8F). Furthermore, tumor tissues from the STEAP3-overexpressing group displayed not only elevated Fe^2+^ and total iron levels but also abnormal mitochondrial morphology characterized by a smaller volume and disrupted cristae compared to the EV group (Figure 7L–N). Additionally, we used a 498-sample NB dataset (GSE49710) from the R2 database to perform a prognostic survival analysis, which showed that patients with NB and higher levels of STEAP3 demonstrated a longer survival time than those with lower expression (Figure S8G).

Furthermore, we investigated whether STEAP3 regulates the ferroptosis triggered by DMAMCL in *MYCN*-amplified NB cells. The results of western blotting revealed that DMAMCL treatment led to a decrease in STEAP3 expression in *MYCN*-amplified BE2 cells (Figure S9A). However, in BE2 cells, *STEAP3* overexpression not only failed to block but also enhanced DMAMCL-induced cell death, while its knockdown had no effect on cell viability (Figure S9B-E). In summary, these results demonstrated that STEAP3 mediated DMAMCL-induced ferroptosis in *MYCN*-nonamplified NB cells but not in *MYCN*-amplified NB cells.

## 4. Discussion

In this study, we demonstrated that DMAMCL induces ferroptosis in NB both *in vitro* and *in vivo*, and the molecular mechanisms of DMAMCL-induced ferroptosis are associated with *MYCN* expression levels. In *MYCN*-amplified NB cells, DMAMCL bound to KEAP1 and increased the levels of NRF2 in the nucleus, leading to HMOX1 upregulation and subsequent ferroptosis. However, in *MYCN*-nonamplified NB cells, *STEAP3* mediated DMAMCL-induced ferroptosis.

Currently, phase II clinical trials of DMAMCL therapy for glioblastoma are underway in China and Australia [8,12]. DMAMCL has demonstrated antitumor efficacy against multiple malignancies, including rhabdomyosarcoma, hepatocellular carcinoma, pituitary tumor, and primary central nervous system lymphoma [11,29–31]. Studies have demonstrated that the antitumor mechanisms of DMAMCL involve apoptosis, autophagy, and cell differentiation [11,32]. Yang et al. reported that MCL induces various forms of cell death, including autophagy, paraptosis, and ferroptosis, in pancreatic and colon cancer via the MAPK signaling pathway [33]. Our study identified that ferroptosis is the predominant form of cell death induced by DMAMCL in NB. Chen et al. demonstrated that the ferroptosis-related gene signature showed significant prognostic value for overall survival in NB patients [34]. A study revealed that blocking selenium/selenocysteine uptake mechanisms via LRP8 is a selective and safe strategy to induce ferroptosis in *MYCN*-amplified NB cells, demonstrating a novel therapeutic approach against this aggressive tumor subtype [35]. Additionally, Monteleone et al. reported that targeting SLC7A11 with either Sulfasalazine or protein kinase Cα potentiates Etoposide sensitivity in therapy-resistant NB cancer stem cells through ferroptosis induction, mediated by GSH depletion and LPO stimulation [26]. Similarly, we observed that DMAMCL induces GSH depletion and elevated LPO levels, suggesting its potential role as a ferroptosis inducer in augmenting the efficacy of combined antitumor therapies. Furthermore, Luo et al. reported that MCL bound to KEAP1, inhibited KEAP1/NRF2 complex formation, and enhanced NRF2 nuclear translocation, which subsequently suppressed ferroptosis in macrophages [36]. In contrast, our study demonstrated that in *MYCN*-amplified NB cells, NRF2 nuclear translocation driven by the similar MCL-KEAP1 binding interaction upregulates HMOX1 expression and ultimately induces ferroptosis. The underlying mechanism may be that the overexpression of HMOX1 promotes heme degradation, generating large amounts of Fe^2+^. Given that MYCN‑high NB cells inherently possess elevated Fe^2+^ levels and are therefore highly sensitive to ferroptosis [37], the pro-oxidative effect resulting from increased HMOX1 expression outweighs its antioxidant capacity in this specific cellular context. This imbalance ultimately induces ferroptosis. In macrophages, NRF2 activation upregulates ferroptosis suppressors such as GPX4 and SLC7A11, thereby inhibiting ferroptosis. These contrasting findings suggest that MCL–KEAP1 binding can produce diametrically opposite regulatory effects on ferroptosis depending on the cellular context.

Additionally, our previous findings demonstrated DMAMCL-induced apoptosis in NB cells [15]. In this study, KEGG pathway enrichment analysis of RNA-seq data also revealed significant enrichment of apoptosis pathways. This indicates that DMAMCL exerts poly-mechanistic cytotoxic effects in NB, potentially involving coordinated activation of both ferroptosis and apoptosis. Emerging studies reveal a regulatory interplay between ferroptosis and apoptosis, with ROS-mediated lipid peroxidation serving as a critical mediator in both cell death pathways [38]. Current research indicates that while NADPH oxidase contributes to apoptotic processes, its ROS-generating function also triggers ferroptosis through lipid peroxidation activation [39–41]. Furthermore, experimental evidence demonstrates that classical ferroptosis inducers like erastin can initiate caspase-9-dependent mitochondrial apoptosis in tumor cells [42]. These mechanistic overlaps collectively highlight the intricate crosstalk between ferroptosis and apoptotic pathways. While current evidence suggests that DMAMCL induced both ferroptosis and apoptosis in NB, the intricate molecular mechanisms of DMAMCL-induced ferroptosis and apoptosis in NB, along with the interplay between these two cell death pathways, require further investigation.

HMOX1, the rate-limiting enzyme in heme catabolism, catalyzes the degradation of heme into biliverdin IXα, CO, and Fe^2+^ [43]. Zhou et al. reported that IFI16 activates HMOX1 transcription to inhibit ferroptosis in Glioblastoma multiforme cells [44]. In contrast, other studies reported that Dihydroartemisinin reduces iron accumulation in microglia via the HIF1A/HMOX1 pathway [45]. Ni et al. demonstrated that shikonin-cisplatin combination therapy induces ferroptosis in ovarian cancer via *HMOX1* upregulation [46]. Similarly, Arnicolide C and Dehydrocostus lactone have been shown to trigger ferroptosis by targeting HMOX1 in liver cancer [47,48]. Interestingly, Yu et al. found that although Acevaltrate significantly upregulates HMOX1 expression in colorectal cancer cells, HMOX1 does not mediate the drug‑induced ferroptosis. Instead, ferroptosis is triggered by targeting other pathways such as PCBP1/PCBP2 [49]. Similarly, in our study, although DMAMCL upregulates HMOX1 in both *MYCN*‑amplified and non-amplified NB cells, HMOX1 only mediated DMAMCL-induced ferroptosis in MYCN‑high cells. In *MYCN*‑nonamplified cells, ferroptosis depends mainly on the upregulation of STEAP3, a key extracellular iron‑uptake gene. This cell‑type‑specific response may stem from distinct basal iron‑metabolism states. MYCN‑high NB cells exhibit elevated iron‑transporter expression and a heightened baseline of extracellular iron import [37], rendering them sensitive to further increases in endogenous ferrous iron via HMOX1. In contrast, in *MYCN*‑nonamplified NB cells, upregulating extracellular iron uptake through STEAP3 serves as the predominant trigger for ferroptosis. Moreover, studies have demonstrated that MYCN coordinately upregulates multiple components of the system Xc^-^/GSH pathway, mediates cysteine addiction, and sensitizes NB cells to ferroptosis [50,51]. *MYCN*-amplified NB cells rely on system Xc^-^ to counteract ROS derived from iron metabolism. These findings establish that MYCN-high NB cells exist in a primed state characterized by elevated iron flux, increased oxidative burden, and dependence on the system Xc^-^/GSH pathway for survival. Within this sensitive state, DMAMCL upregulates the expression level of HMOX1, leading to the release of more free iron. The already weak antioxidant defense in MYCN-high cells cannot handle this extra iron surge, resulting in ferroptosis. Therefore, the role of HMOX1 in mediating DMAMCL‑induced ferroptosis in NB cells depends on the MYCN expression background.

STEAP3 is involved in the reduction of intracellularly transported Fe^3+^ to Fe^2+^, thereby playing a critical role in maintaining cellular iron homeostasis [52]. Recent studies reported that the upregulation of *STEAP3* drives iron overload in cells, thereby inducing ferroptosis [53,54]. Similar to these studies, we observed that DMAMCL-induced upregulation of *STEAP3* promotes intracellular Fe^2+^ accumulation and LPO, thereby driving ferroptosis in *MYCN*-nonamplified NB cells. Furthermore, in MYCN-nonamplified AS cells, we found that combined knockdown of STEAP3 and HMOX1 did not significantly affect DMAMCL-induced cell death compared with STEAP3 knockdown alone (Figure S10), indicating that the role of STEAP3 in mediating DMAMCL-induced ferroptosis in this cell type is independent of the NRF2/HMOX1 pathway. Emerging evidence indicates that elevated STEAP3 expression correlates with reduced survival rates in patients with triple-negative breast cancer (TNBC) [55]. Another study reported that elevated STEAP3 levels in gliomas exhibit a significant inverse correlation with overall survival rates, while functionally acting as a critical oncogenic driver promoting tumor progression [56]. However, high levels of STEAP3 were associated with a higher survival rate in NB patients. We demonstrated that overexpression of *STEAP3* suppressed *MYCN*-nonamplified AS xenograft tumor growth and prolonged the survival of mice. This indicates that the role of STEAP3 varies across different cancer types, and it can serve as a potential therapeutic target in *MYCN*-nonamplified NB.

## 5. Conclusion

In summary, we demonstrated that DMAMCL induces ferroptosis in NB via a molecular mechanism that depends on *MYCN* expression levels. Specifically, DMAMCL increases the levels of NRF2 in the nucleus by binding to KEAP1, thereby upregulating HMOX1 expression and ultimately inducing ferroptosis in *MYCN*-amplified NB cells, whereas it primarily promotes ferroptosis via STEAP3 upregulation in *MYCN*-nonamplified NB cells. This study uncovered a novel molecular mechanism of DMAMCL-induced ferroptosis and provides a promising therapeutic target for NB with different *MYCN* expression levels.

## Supplementary Material

ARRIVE guidelines.pdf ARRIVE guidelines.pdf

Supplemental table 1_clean version.docx Supplemental table 1_clean version.docx

Supplementary figures_clean version.docx Supplementary figures_clean version.docx

## Data Availability

All supporting data are included within the article, and all the data generated in this article are available from the first author on reasonable request.
