# Supplementary material for: DMAMCL induces ferroptosis in neuroblastoma by targeting HMOX1 in MYCN-amplified subtypes whereas targeting STEAP3 in MYCN-nonamplified subtypes
Source: Redox Rep. 2026 Jul 13;31(1):2702136. doi: 10.1080/13510002.2026.2702136 (PMC13371481; doi:10.1080/13510002.2026.2702136)
Supplement: Supplemental table 1_clean version.docx [file YRER_A_2702136_SM5875.docx]

**Supplemental table**

| **Gene name/**  **Accession code**  **(Human)** | **Forward primer (5' to 3')** | **Reverse primer (5' to 3')** |  |
| --- | --- | --- | --- |
| *GAPDH*  NM_002046.7 | CATGTTGCAACCGGGAAGGA | CAGGAGCGCAGGGTTAGTC |  |
| *HMOX1*  NM_002133.3 | ATTTCAGAAGGGCCAGGTGA | GGAAGTAGACAGGGGCGAAGA |  |
| *AKR1C1*  NM_001353.6 | GCCAGCACACTAAGCCTATCTACAG | GCAGTTCTTCATCCTCCAGCCATC |  |
| *GPX3*  NM_002084.5 | GGGCAATCCCCAGATGGACT | TCAATGGTGAGGGCTCCGTA |  |
| *GCLM*  NM_002061.4 | CAGCGAGGAGGAGTTTCCAG | GAACAGGCCATGTCAACTGC |  |
| *NQO1*  NM_000903.3 | CTGAAAGGCTGGTTTGAGCGA | TGTCCCCGTGGATCCCTT |  |
| *SLC7A11*  NM_014331.4 | ATGGGACAAGAAACCCAGGTG | TCCCTATTTTGTGTCTCCCCTTG |  |
| *AKR1C3*  NM_003739.6 | TAGCCAGCTGAGTGACAGTGAT | AATCCCAGGACAGGCATGAAGTG |  |
| *BLVRB*  NM_000713.3 | AATGACCTCAGTCCCACGAC | TTGTCCACACCATGAGCCTT |  |
| *OSGIN1*  NM_182981.3 | TGGGGTCAAGAGGAGTAGGG | GCAGCTTTCCCCACAAATCC |  |
| **Table S1. The sequence information of the primers used in this study.** This table lists the forward and reverse primers (5' to 3') for the indicated human genes. All primers were checked for specificity and used in qRT-PCR. | | |  |
|  |  |  |  |
|  |  |  |  |
|  |  |  |  |
|  |  |  |  |
