# Supplementary material for: DMAMCL induces ferroptosis in neuroblastoma by targeting HMOX1 in MYCN-amplified subtypes whereas targeting STEAP3 in MYCN-nonamplified subtypes
Source: Redox Rep. 2026 Jul 13;31(1):2702136. doi: 10.1080/13510002.2026.2702136 (PMC13371481; doi:10.1080/13510002.2026.2702136)
Supplement: Supplementary figures_clean version.docx [file YRER_A_2702136_SM5876.docx]

**Supplement figures**


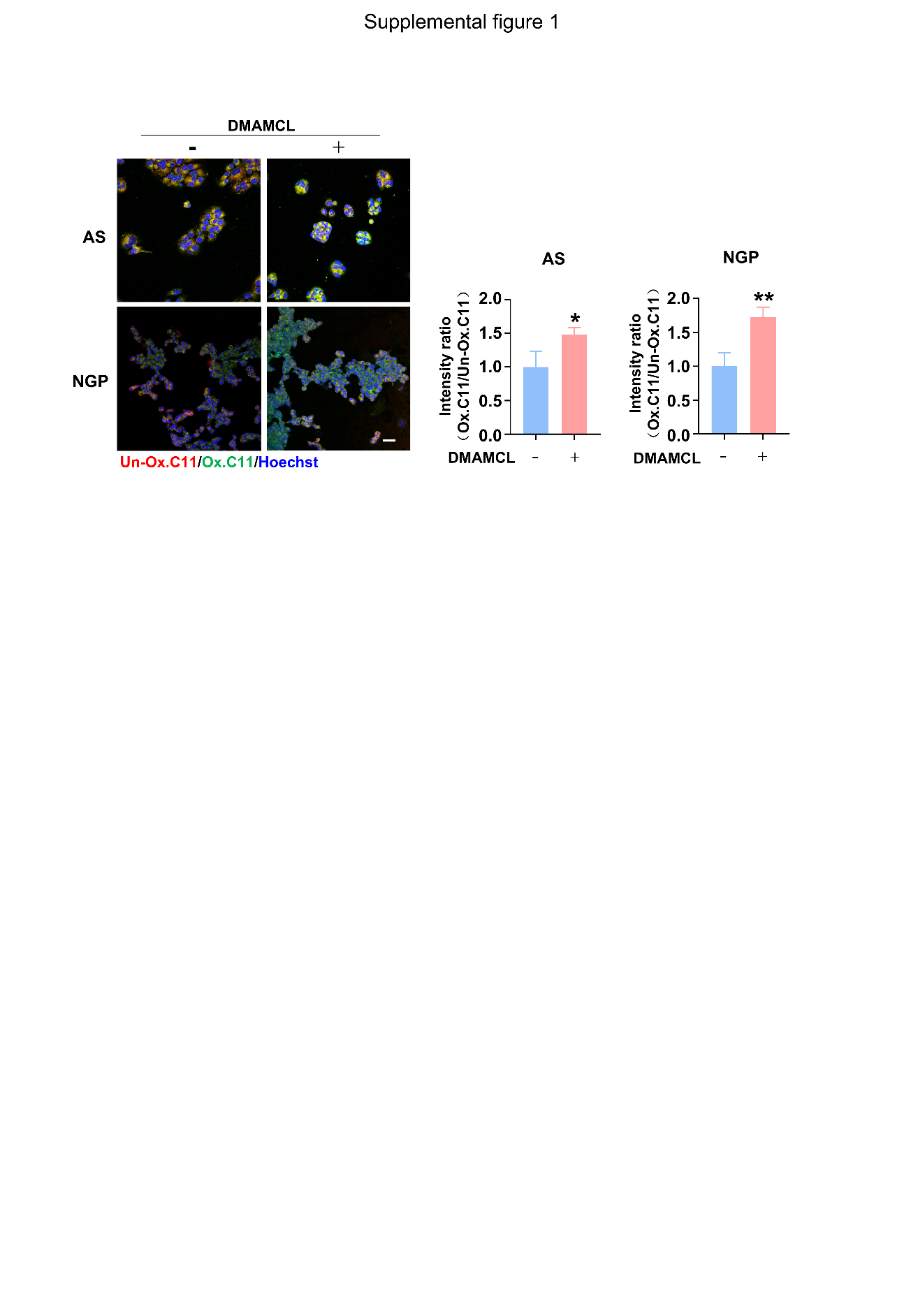
 **Fig. S1 DMAMCL treatment increased the LPO levels in NB cells.** LPO levels of AS and NGP after DMAMCL treatment for 8 h were detected using BODIPY-C11 and observed using confocal microscope, scale bar, 50 μm. Data were represented as mean ± SD, n = 3 independent biological replicates, **P* < 0.05, ***P* < 0.01, non-significant (ns).


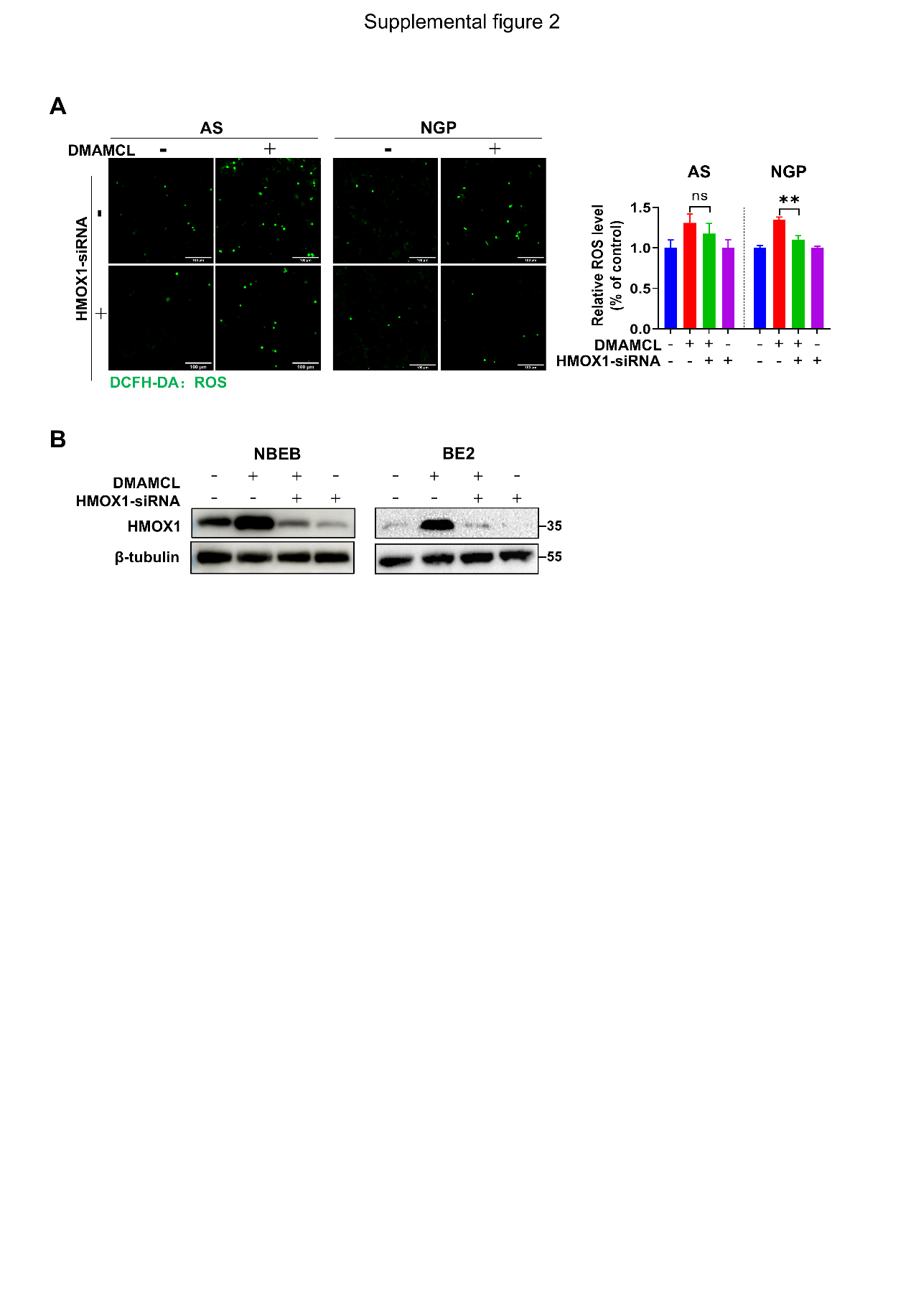


**Fig. S2 ROS levels and HMOX1 expression after *HMOX1* knockdown combined with DMAMCL treatment in NB cells.** (A) Fluorescence intensity of ROS (Green) in AS and NGP cells transfected with control-siRNA or *HMOX1*-siRNA followed by 24 h DMAMCL treatment were observed. Quantitative analysis of fluorescence intensity was performed using ImageJ software. Scale bar, 100 μm. (B) HMOX1 protein levels in BE2 and NBEB cells transfected with *HMOX1*-siRNA or control-siRNA, followed by DMAMCL treatment, were detected by Western blotting. Data were represented as the mean ± SD, n = 3 independent biological replicates. ***P* < 0.01, non-significant (ns).


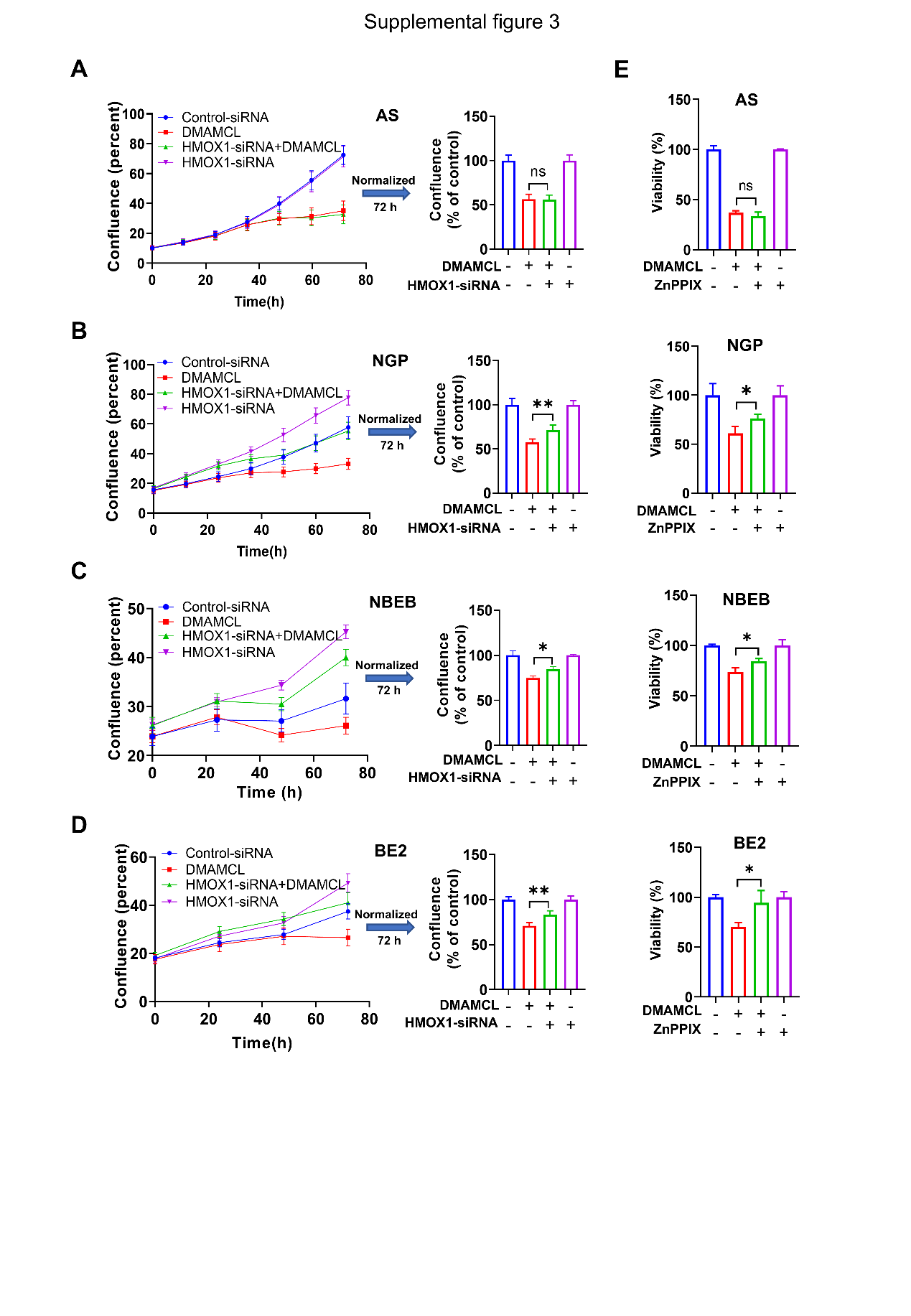


**Fig. S3 Cell confluence and viability in response to DMAMCL after *HMOX1* Knockdown or inhibition.** (A-D) Cell confluence of AS, NGP, NBEB, and BE2 cells transfected with *HMOX1-*siRNA or control-siRNA and followed by DMAMCL treatment. Cell confluence was observed using the IncuCyte ZOOM live cell imaging system and the data were presented as line graph. Endpoint data presented as column graphs and were normalized: DMAMCL-treated group was normalized to the control (set as 100%), *HMOX1-*siRNA + DMAMCL group was normalized to *HMOX1-*siRNA group (set as 100%). Representative images of BE2 and NBEB cells transfected with *HMOX1-*siRNA or control-siRNA followed by DMAMCL treatment. Scale bar, 300 μm. (E) Cell viability of AS, NGP, NBEB, and BE2 cells treated with DMAMCL for 48 h in the presence or absence of ZnPPIX (2.5 μM). Data were represented as the mean ± SD, n = 3 independent biological replicates. **P* < 0.05, ***P* < 0.01, non-significant (ns).


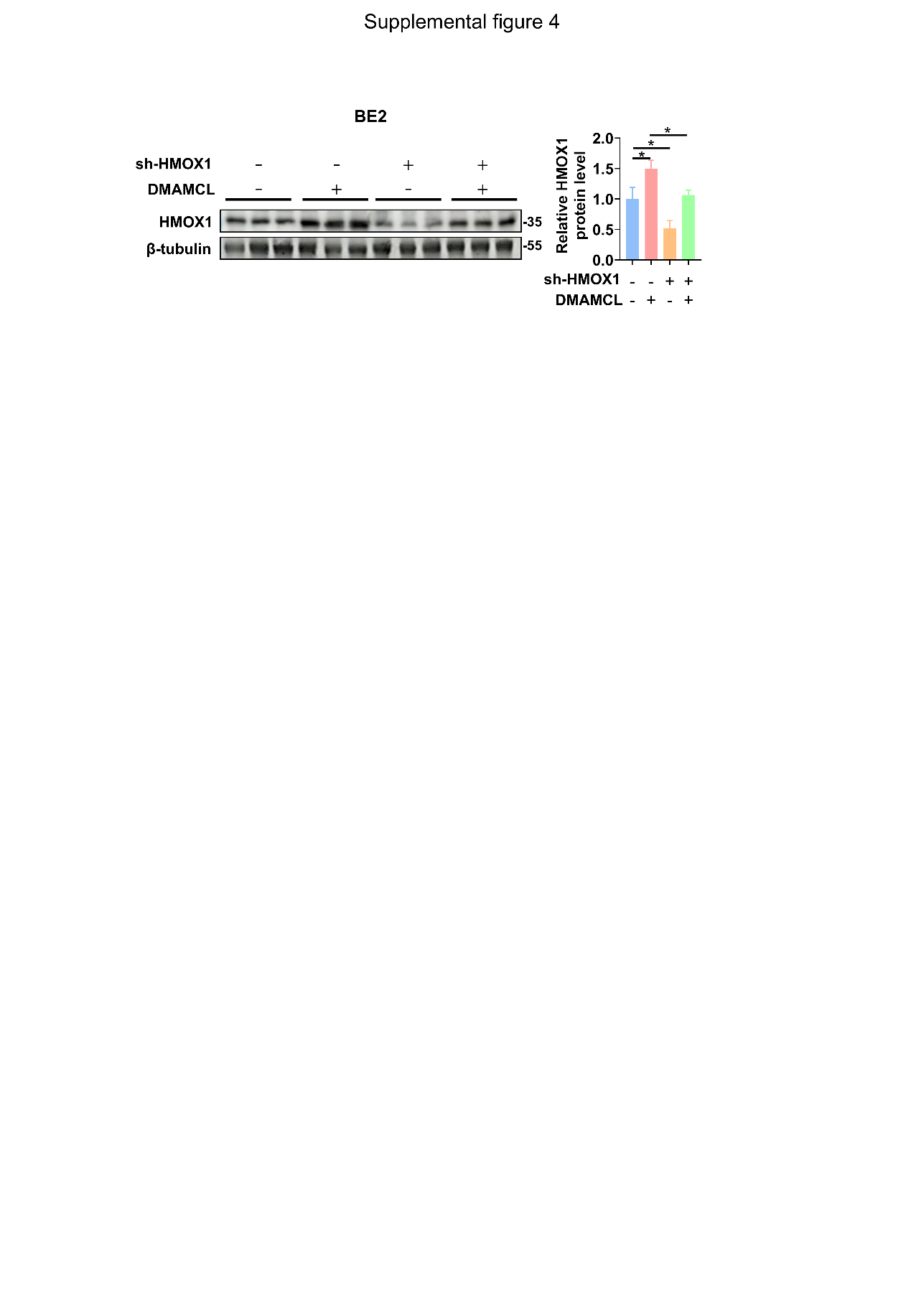


**Fig. S4 Validation of *HMOX1*-shRNA in BE2 Xenograft Tumor Tissues.** Western blotting detected the protein level of HMOX1 in BE2 tumor tissues. The protein band intensities were quantified using ImageJ software, and the results are presented as a histogram. Data were represented as the mean ± SEM, n = 3 independent biological replicates. **P* < 0.05.


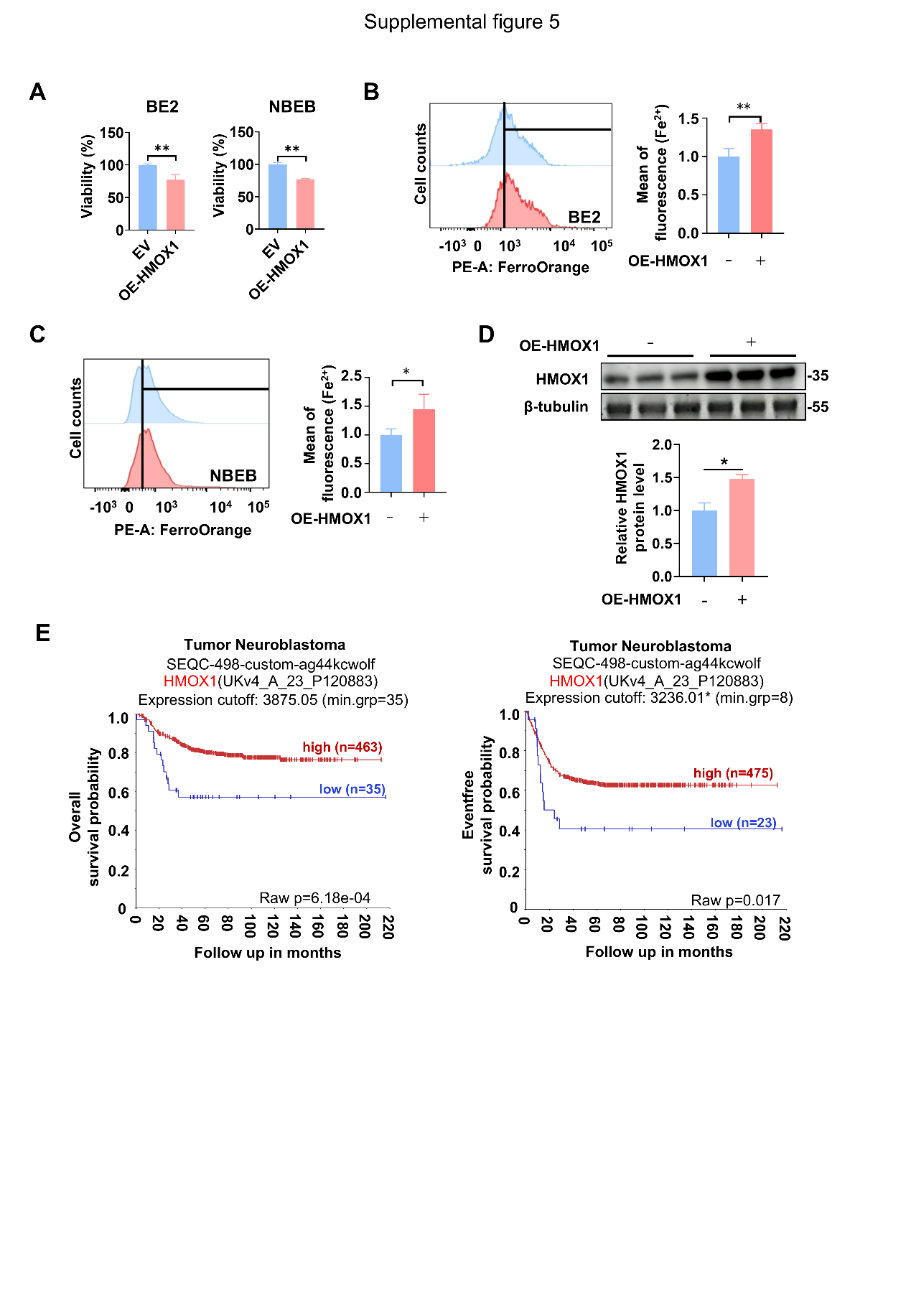


**Fig. S5 The effects of *HMOX1* overexpression in MYCN-high NB cells.** (A) Cell viability was assessed using CCK-8 assays after overexpressing *HMOX1* for 72 h in BE2 and NBEB. (B, C) Fe^2+^ levels of BE2 and NBEB cells after overexpressing *HMOX1* were detected using FerroOrange and quantitatively analyzed by flow cytometry. (D) Western blotting detected HMOX1 protein levels of BE2 tumor tissues and subjected to grayscale analysis of the protein bands using Image J. (E) Kaplan-Meier survival analysis was performed using the R2 database (https://hgserver1.amc.nl/cgi-bin/r2/main.cgi) to assess the prognostic significance of HMOX1. Data were represented as the mean ± SD in (A-C) and as the mean ± SEM for (D), n = 3 independent biological replicates. **P* < 0.05, ***P* < 0.01.


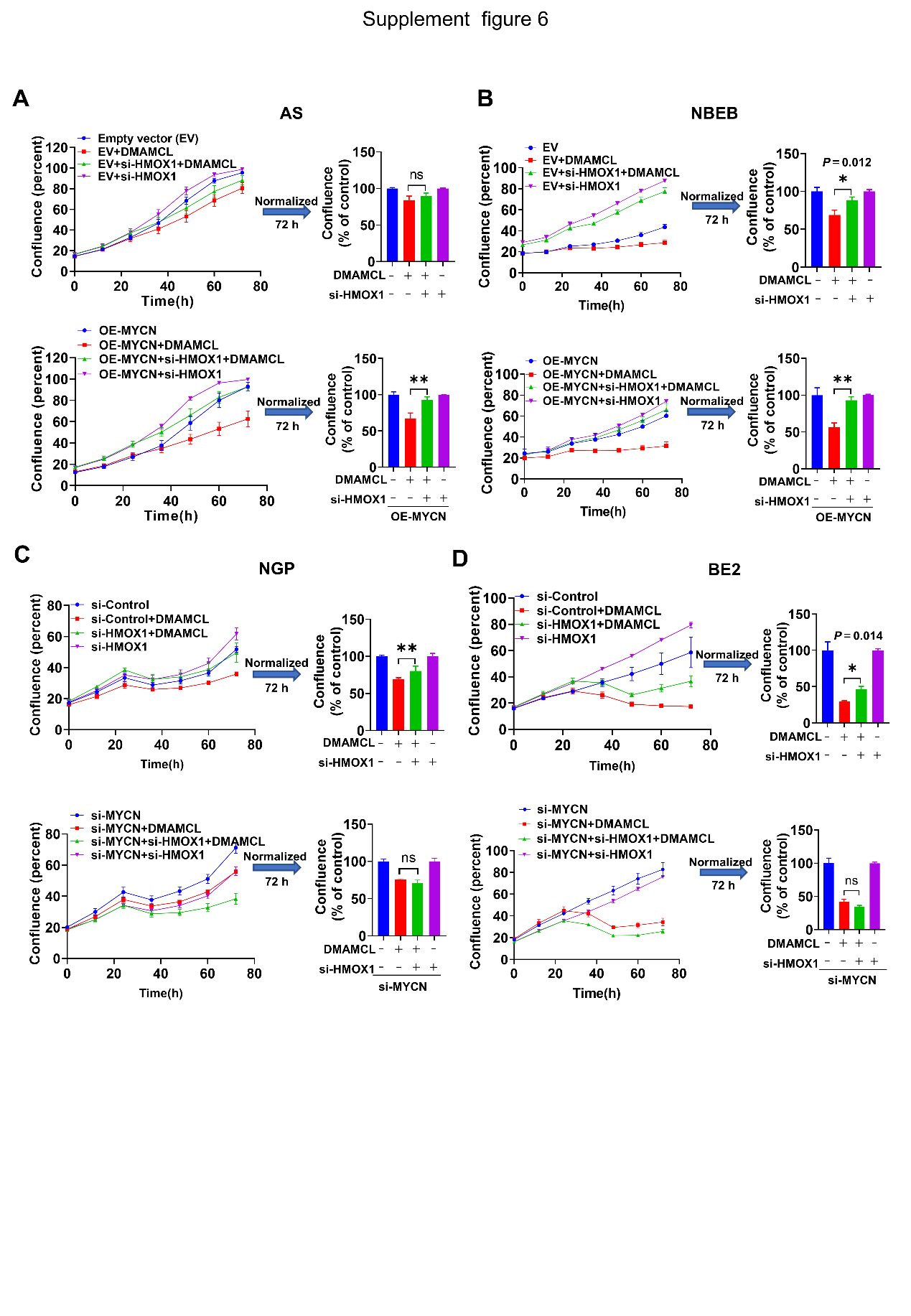


**Fig. S6 After modulating *MYCN* expression levels, the cell confluence after *HMOX1* Knockdown combined to DMAMCL treatment in NB cells.** (A, B) Cell confluence was analyzed between *HMOX1-*siRNA + DMAMCL group and DMAMCL alone treatment group in AS and NBEB cells transfected with or without *MYCN*-overexpressing plasmid. (C, D) Cell confluence was analyzed between *HMOX1-*siRNA + DMAMCL group and DMAMCL alone treatment group in AS and NBEB cells transfected with or without *MYCN*-siRNA in NGP, and BE2 cells. Data in (**A**–**D**) were normalized: DMAMCL-treated group was normalized to the control (set as 100%), *HMOX1*-siRNA + DMAMCL group was normalized to *HMOX1-*siRNA group (set as 100%). Data were represented as the mean ± SD, n = 3 independent biological replicates. **P* < 0.05, ***P* < 0.01, non-significant (ns).


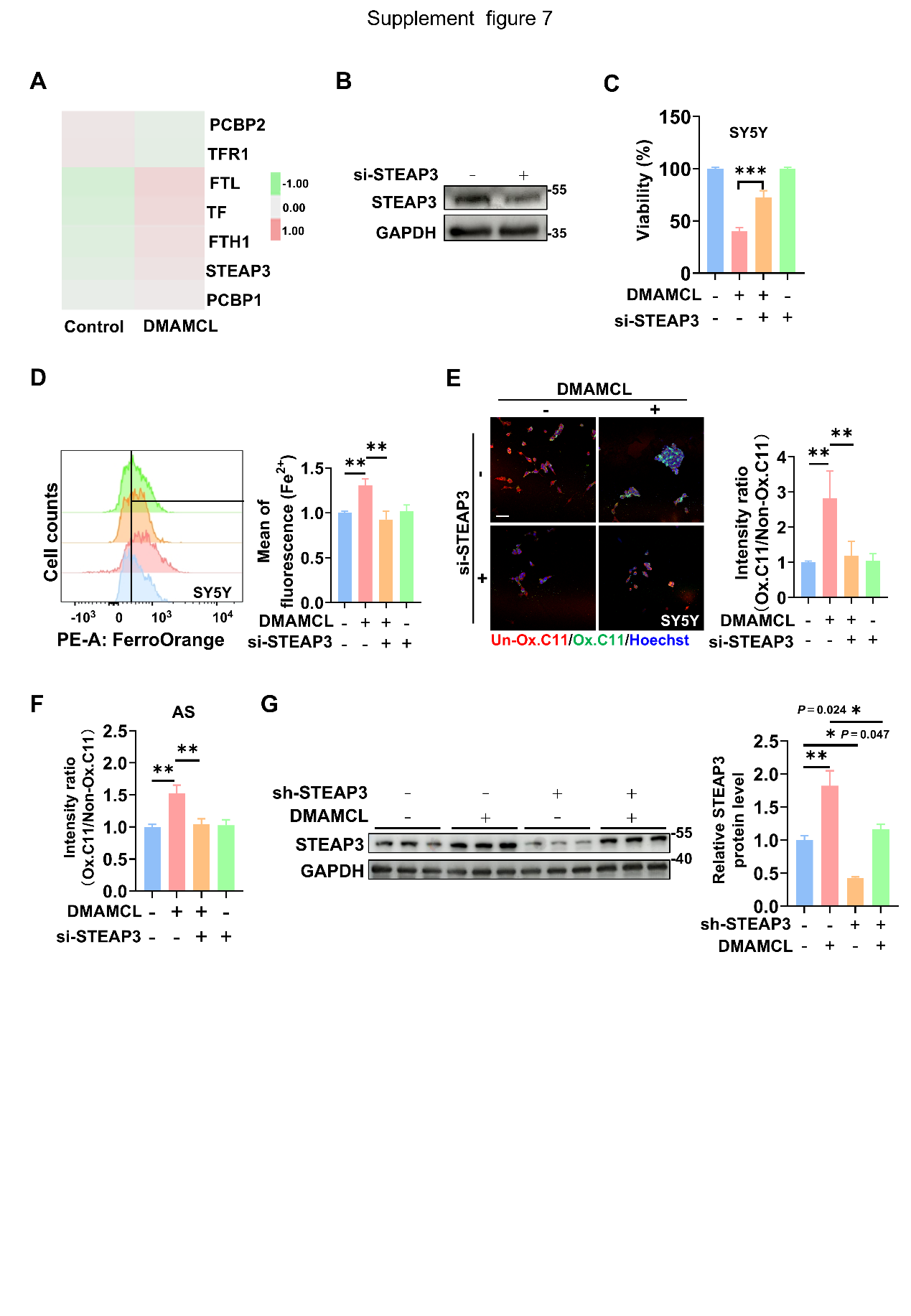


**Fig. S7 STEAP3 mediates DMAMCL-induced ferroptosis in *MYCN*-nonamplified NB cells.** (A) A heatmap of changes in iron metabolism-related genes in AS cells after 12 hours of DMAMCL treatment. (B) Western blotting detected *STEAP3* protein levels after knocking down *STEAP3* in AS cells. (C) Cell viability of SY5Y cells after transfecting with *STEAP3*-siRNA or control-siRNA and followed by DMAMCL treatment. (D) Fe^2+^ levels of SY5Y after transfecting with *STEAP3*-siRNA or control-siRNA and followed by DMAMCL treatment for 24 h were detected using FerroOrange and quantitatively analyzed by flow cytometry. (E) LPO levels of SY5Y after transfecting with *STEAP3*-siRNA or control-siRNA and followed by DMAMCL treatment for 8 h were detected using BODIPY-C11 and observed using confocal microscope. Quantitative analysis of fluorescence intensity was performed using ImageJ software, scale bar, 50 μm. (F) Quantitative analysis of fluorescence intensity for LPO levels in AS was performed using ImageJ software. (G) The protein levels of STEAP3 in AS tumor tissues were detected by Western blot and subjected to grayscale analysis of the protein bands using Image J. Data represent the mean± SD in (C-F) and were represented as the mean ± SEM in (G), n = 3 independent biological replicates. **P* < 0.05, ***P* < 0.01, ****P* < 0.001.


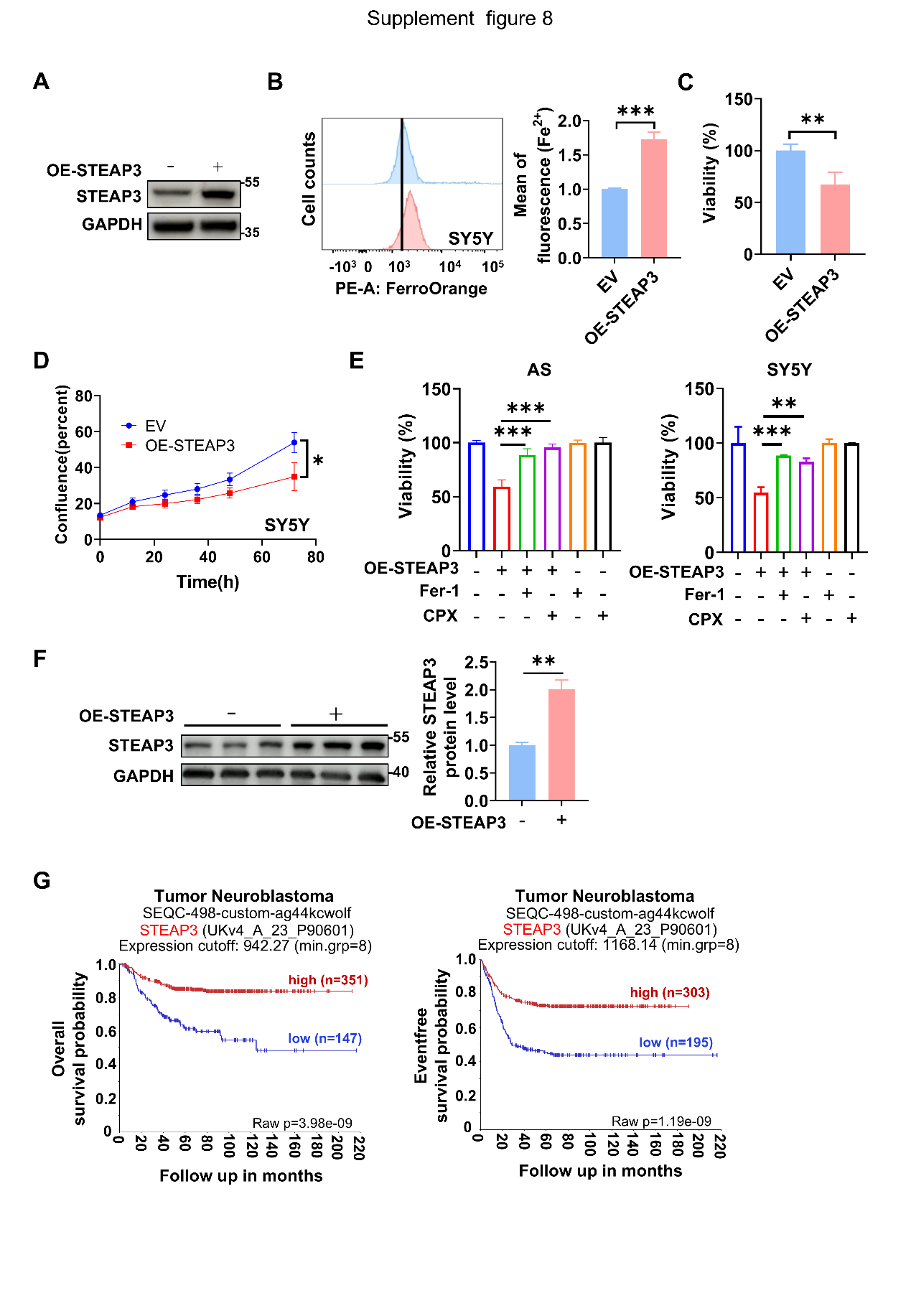


**Fig. S8 The effects of STEAP3 overexpression in *MYCN*-nonamplified NB cells.** (A) Western blotting was used to detect the protein levels of STEAP3 after transfection with the *STEAP3* overexpression plasmid in AS cells. (B) Fe^2+^ levels of SY5Y after overexpressing *STEAP3* were detected using FerroOrange and quantitatively analyzed by flow cytometry. (C) Cell viability of SY5Y after overexpressing *STEAP3* were detected using CCK-8 assays. (D) Cell confluence of SY5Y after overexpressing *STEAP3* was detected using IncuCyte ZOOM live cell imaging system. (E) AS and SY5Y cells overexpressing STEAP3 were treated with Fer-1 (10 μM) or CPX (2.5 μM) for 48 h, and cell viability was assessed by CCK-8 assay. (F) Western blotting detected STEAP3 protein levels of AS tumor tissues and subjected to grayscale analysis of the protein bands using Image J. (G) Kaplan-Meier survival analysis was performed using the R2 database (https://hgserver1.amc.nl/cgi-bin/r2/main.cgi) to assess the prognostic significance of STEAP3. Data were represented as mean ± SD in (B-D) and data were represented as mean ± SEM in (E), n = 3 independent biological replicates. **P* < 0.05, ***P* < 0.01, ****P* < 0.001.

**
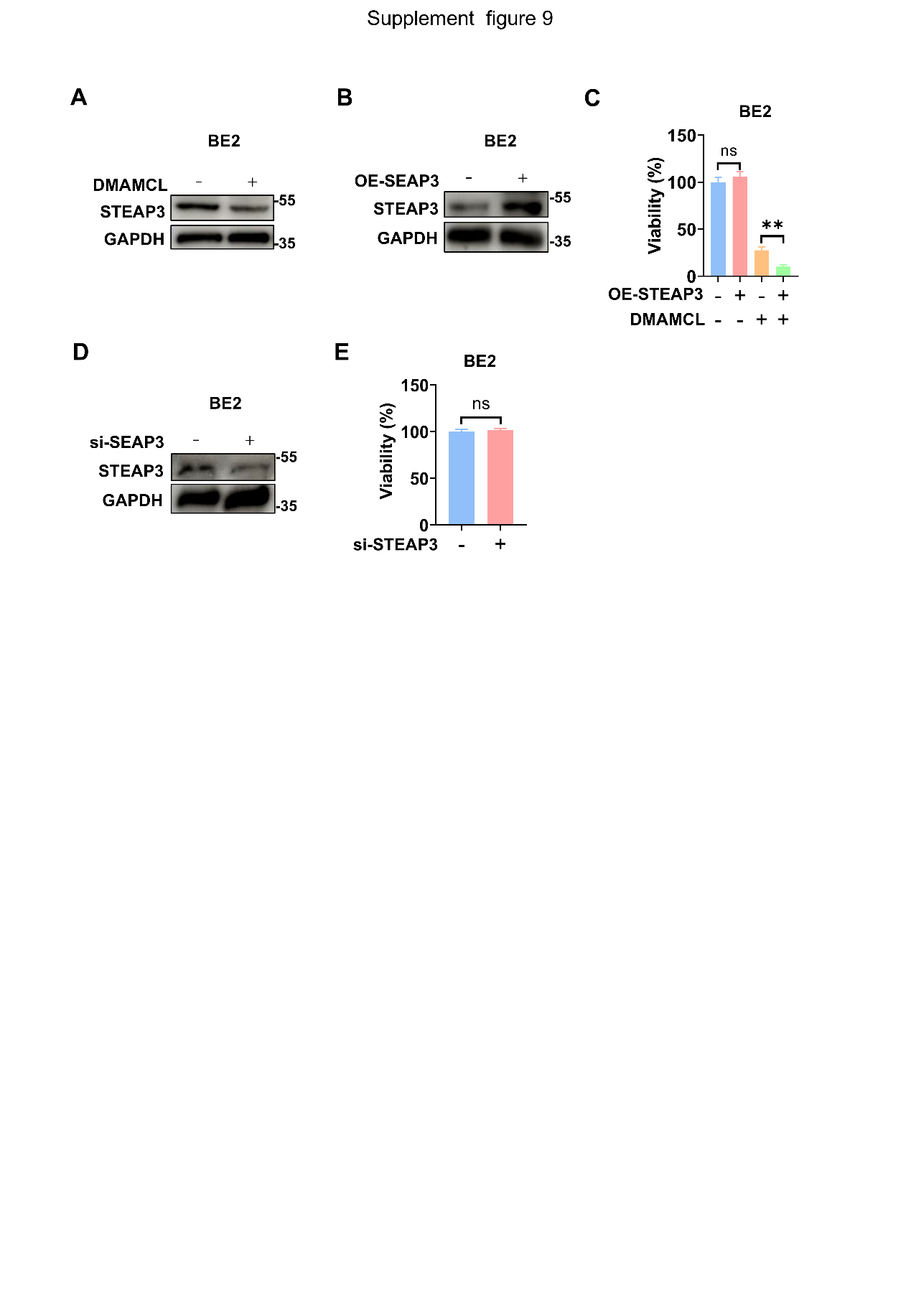
**

**Fig. S9** **STEAP3 does not mediate DMAMCL-induced ferroptosis in MYCN-amplified BE2 cells.** (A) The protein level of STEAP3 in BE2 cells treated with DMAMCL for 6 h was detected by Western blotting. (B) STEAP3 protein levels were measured by Western blot in BE2 cells after overexpression of STEAP3. (C) BE2 cells were subjected to STEAP3 overexpression followed by DMAMCL treatment, and cell viability was measured by CCK-8 assay. (D) STEAP3 protein levels were measured by Western blot in BE2 cells after knockdown of STEAP3. (E) BE2 cells were transfected with *STEAP3*-siRNA, and cell viability was assessed by CCK-8 assay. Data were represented as mean ± SD, n = 3 independent biological replicates, ***P* < 0.01, non-significant (ns).


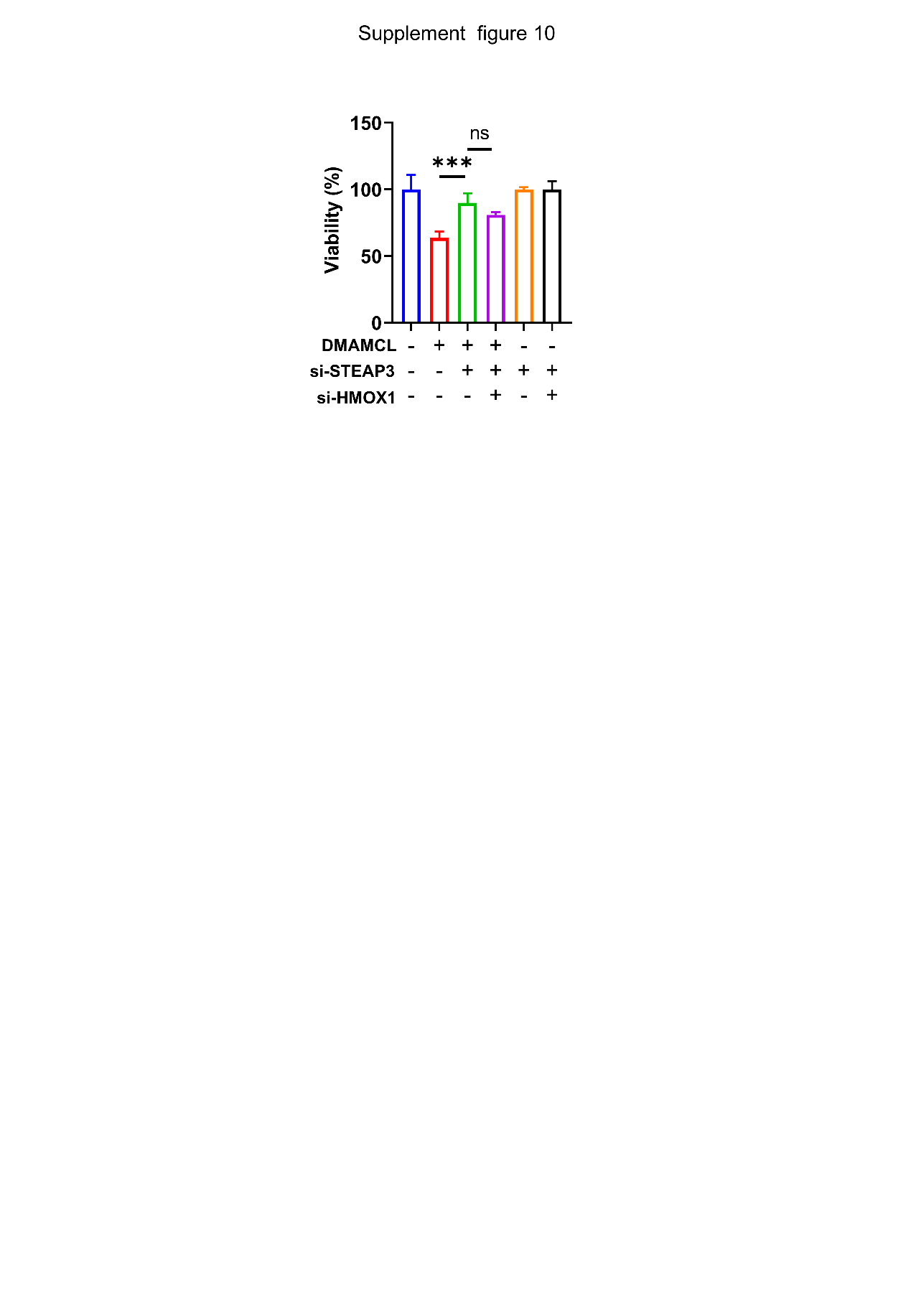


**Fig. S10**. **The role of STEAP3 in mediating DMAMCL-induced ferroptosis is independent of the NRF2–HMOX1 pathway in *MYCN*-nonamplified AS cells.** AS cells were transfected with HMOX1-siRNA or STEAP3-siRNA, respectively, and then treated with DMAMCL for 48 h. Cell viability was assessed by CCK-8 assay. Data were represented as mean ± SD, n = 3 independent biological replicates, ****P* < 0.001, non-significant (ns).
